# Exploring *α*-Terpineol–Antibiotic Synergistic Combinations as an Environmentally Safer Antimicrobial Strategy for Freshwater Systems

**DOI:** 10.3390/antibiotics15090929

**Published:** 2026-09-19

**Authors:** Diego Ballestero, Antonio Valenzuela, Pedro Rodríguez-López, Cristina Gan, Laura Botello-Morte, Elisa Langa, María Rosa Pino-Otín

**Affiliations:** Faculty of Health Sciences, Universidad San Jorge, Autovía A23 Zaragoza-Huesca km. 299, 50830 Villanueva de Gállego, Spain; dballestero@usj.es (D.B.); avalenzuela@usj.es (A.V.); prodriguezl@usj.es (P.R.-L.); cgan@usj.es (C.G.); lbotello@usj.es (L.B.-M.); elanga@usj.es (E.L.)

**Keywords:** *Aliivibrio fischeri*, Antibiotics, Antimicrobial Resistance, *Daphnia magna*, ecotoxicity, freshwater microbial communities, one health, terpineol

## Abstract

**Background:** This study evaluated the ecotoxicity of five synergistic antimicrobial combinations of terpineol-antibiotic (SACTA), developed to reduce antibiotic (ABX) doses while maintaining antimicrobial efficacy. The ABXs tested were gentamicin (GTM), chloramphenicol (CHL), tetracycline (TC), and florfenicol (FF). **Methods:** Median effective concentrations (*EC*_50_) were determined using *Aliivibrio fischeri* and *Daphnia magna*. In addition, the effects of SACTAs on the metabolic profile of an indigenous riverine microbial community were assessed using Biolog EcoPlates™. **Results:** *α*-Terpineol (TER) alone showed *EC*_50_ values of 21.49 and 17.64 µg/mL for *D. magna* and *A. fischeri*, respectively. The toxicity of SACTAs varied depending on the antimicrobial combination, exhibiting synergistic, additive, or antagonistic effects. Among the tested combinations, TER-CHL was the most toxic with *EC*_50_ values of 7.84 µg/mL for *D. magna* and 12.75 µg/mL for *A. fischeri*. In contrast, TER-FF and TER-GTM, with lower TER proportions, were the least toxic, with *EC*_50_ values exceeding 30 µg/mL for *D. magna* and 60 µg/mL for *A. fischeri*. Although SACTAs generally showed higher toxicity than ABXs tested individually, their *EC*_50_ values remained below the minimum inhibitory concentrations (MICs) determined in single-species antimicrobial assays. In contrast, riverine microbial communities displayed greater resilience, with only additive or antagonistic effects observed. Among the tested combinations, TER-FF produced the strongest effects on the microbial community, reducing metabolic fitness and decreasing the utilization of carbohydrates and polymers. Importantly, all SACTAs exhibited toxicity values equal to or lower than those of ABXs tested alone at their respective MICs, based on changes in community growth and metabolic profiles. **Conclusions:** Overall, these findings suggest that SACTAs may represent a promising strategy for reducing ABX use while minimizing additional ecotoxicological risks to freshwater ecosystems.

## 1. Introduction

The widespread use of antibiotics (ABXs) has considerably increased in recent decades [1], resulting in the continuous release of these compounds and their metabolites into aquatic environments [2]. This environmental contamination raises several concerns, including ecological damage, the selection and spread of antimicrobial resistance (AMR) and the dissemination of antimicrobial resistance genes (ARGs) [3,4,5]. Consequently, there is an urgent need to develop alternative strategies capable of reducing ABX consumption while maintaining therapeutic efficacy and minimizing environmental impact.

One promising approach involves combining conventional ABXs with natural bioactive compounds. In particular, monoterpenes have attracted considerable attention because of their antimicrobial properties and their potential to enhance ABX activity. In a previous study, we demonstrated that synergistic antimicrobial combinations of *α*-terpineol (TER) and ABXs (SACTAs) maintained antibacterial efficacy while reducing the minimum inhibitory concentration (MIC) required for the bacterial growth inhibition [6]. Although these findings highlight the potential of SACTAs as ABX-sparing therapies, their environmental implications remain largely unexplored.

Terpineols (C_10_H_18_O) are monocyclic monoterpene tertiary alcohols naturally present in essential oils from several plants such as *Pinus pinaster*, *Origanum majorana*, *Salvia sclarea*, *Thymus caespititius*, and *Narcissus poeticus* [7]. TER has numerous applications in the pharmaceutical industry [8] and exhibits well-documented antimicrobial activity, particularly against bacterial and fungal pathogens [9,10,11,12]. Previous studies have shown that TER can enhance the efficacy of conventional ABXs, allowing reductions in the therapeutic concentrations required for bacterial inhibition. Examples include its synergistic activity with vancomycin against *Clostridioides difficile* [13] and with cefixime against *Klebsiella pneumoniae* [14]. Based on these properties, our group previously identified five SACTAs involving TER combined with gentamicin (GTM), tetracycline (TC), chloramphenicol (CHL), or florfenicol (FF) [6]. These ABXs are extensively used in both human and veterinary medicine, including livestock production.

In addition to its pharmaceutical applications, TER is widely used in cosmetics, soaps, and air fresheners, and other consumer products, with an annual global production exceeding 1000 tons [15]. It is also employed as a flavouring agent in food systems [16] and as an insect attractant in biological pest control programs [17]. The increasing production and widespread use of TER have contributed to its release into the environment. Although wastewater treatment processes reduce TER concentrations, the compound remains detectable in treated effluents at concentrations ranging from 2.5·10^−5^ to 7.61·10^−3^ µg/mL [18]. Its persistence in aquatic systems has led to its classification as a micropollutant in Europe [19], and environmental monitoring studies have reported concentrations of 8.5·10^−5^ µg/L in the Nechkar River (Germany) [20].

Although TER is classified as a Generally Recognised As Safe (GRAS) compound within established exposure limits [21,22], its environmental effects remain insufficiently characterised. Due to its water solubility, TER can readily interact with aquatic organisms, raising concerns regarding its ecotoxicological impact. Available evidence indicates that TER may affect a range of aquatic organisms, including invertebrates, algae, and fish, impairing growth, reproduction, and survival [23]. For example, TER acute toxicity has been reported in *Artemia salina*, with median effective concentration (*EC*_50_) values of 0.068 µg/L and 0.076 µg/L after 24 and 48 h of exposure, respectively [24]. Similarly, Da Silva et al. [25] reported toxicity (*EC*_50_ = 49.6 µg/mL) in *Artemia* spp. exposed to TER-rich extracts of *Lippia schomburgkiana*. In algae, Chen et al. [26] registered that TER inhibited growth and induced cell death in *Chlamydomonas reinhardtii* through DNA damage and apoptosis-related mechanisms. Furthermore, Stroh et al. [27] demonstrated TER toxicity after 96 h of exposure in *Oncorhynchus* species (f. *Salmonidae*), reporting *EC*_50_ values of 6.3·10^−6^ µg/L for *O. kisutch* and 6.6·10^−6^ µg/L for *O. mykiss*.

In contrast, the occurrence and ecotoxicity of ABXs in aquatic environments have been well documented. GTM, CHL, TC, and FF have been detected in wastewater effluents across Europe [28] and in urban wetlands in China, where they may pose risks to non-target organisms [29]. GTM concentrations ranging from 0.4 to 7.6 µg/mL have been reported in wastewater systems [30,31]. Likewise, Song et al. [32] detected TC concentrations of up to 0.551 µg/L and FF concentrations exceeding 2 µg/L in freshwater aquaculture ponds near Tai Lake, China. CHL has been detected in Tunisian seawater at concentrations reaching 15.6 µg/L, while FF concentrations as high as 18.4 µg/L have also been reported [30].

Numerous studies have demonstrated that these ABXs can exert toxic effects on non-target organisms, including bioindicator species such as *Daphnia magna* or *Aliivibrio fischeri* [33]. Adverse effects have also been reported in fish and algae, including impaired growth and reproduction, even at relatively low concentrations [34,35]. Beyond direct toxicity, ABXs may alter the structure and metabolic activity of aquatic microbial communities, thereby compromising ecosystem functioning and resilience [36].

Despite increasing knowledge regarding the individual effects of TER and ABXs, the ecological impact of their synergistic combinations remains largely unknown. Studies addressing the effects of TER-ABX mixtures on non-target organisms are scarce, and investigations at the microbial community level are, to the best of our knowledge, currently unavailable. This lack of information limits a comprehensive assessment of the environmental risks associated with these promising antimicrobial strategies.

To address this knowledge gap, the present study adopted an integrated approach to evaluate the ecotoxicity of SACTAs and their individual components in aquatic environments. The antibiotics included in the SACTAs were specifically selected for this ecotoxicological assessment because they belong to different chemical classes and modes of action (aminoglycosides, phenicols and tetracyclines) that are particularly relevant for the development and dissemination of antimicrobial resistance, have documented environmental occurrence in freshwater systems, and have already demonstrated synergistic activity with TER at substantially reduced MICs in our previous work. Accordingly, toxicity was first assessed in two non-target model organisms, *A. fischeri* and *D. magna*, using dose–response assays coupled with regression modelling. Second, the temporal effects of these compounds on the metabolic fingerprint of a natural riverine microbial community were investigated using a previously characterised microbiome and complemented with 16S rRNA gene metabarcoding data.

## 2. Results and Discussion

### 2.1. TER and ABXs Acute Toxicity in D. magna and A. fischeri

The results showed that TER alone was more toxic to both indicators than any of the four ABXs tested, as reflected by the corresponding *EC*_50_ values (Table 1).

**Figure 1:**
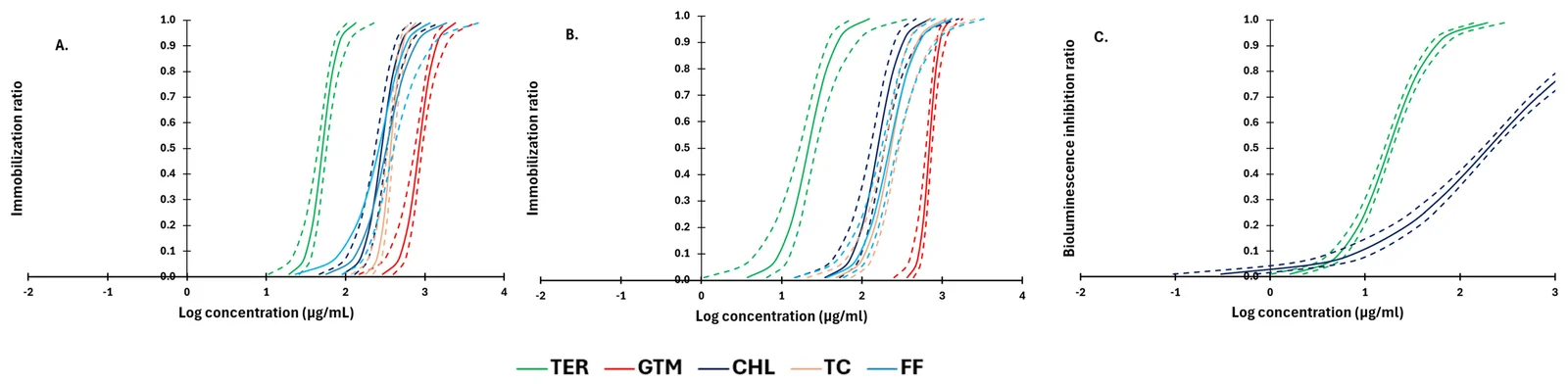
Dose–response curves for *D. magna* after 24 h (**A**) and 48 h (**B**), and for *A. fischeri* after 30 min (**C**) post-exposure to gentamicin (GTM), chloramphenicol (CHL), tetracycline (TC), florfenicol (FF) and terpineol (TER). Solid lines represent the fitted mean response based on experimental data, while dashed lines indicate the 95% confidence intervals. Notably, for *A. fischeri*, TC was excluded due to methodological incompatibility, whereas GTM and FF were omitted because their *EC*_50_ values exceeded the concentration range tested in this study. Complete data on logit fitting are provided in Table S1.

As shown in Figure 1, the toxicity of all compounds towards *D. magna* increased with exposure time, with TER exhibiting an almost two-fold increase in toxicity, suggesting a rapid mode of action. Among the ABXs, CHL was the most toxic compound, followed by FF and TC, which showed similar toxicity values, while GTM was the least toxic.

For *A. fischeri*, only CHL and TER were further evaluated, because the *EC*_50_ values of GTM and FF exceeded 1000 μg/mL (Table 1, Figure 1). In the case of TC, *EC*_50_ values could not be determined because its intrinsic coloration interfered with the fluorescence-based detection method.

To the best of our knowledge, this is the first study reporting the ecotoxicity of TER in these two aquatic bioindicators. Previous studies have shown that other antimicrobial phytochemicals, including hydroquinone, tannic acid, and eugenol, exhibit higher toxicity than many conventional ABXs. Reported *EC*_50_ values range from 0.142 to 32.03 μg/mL for *D. magna* after 24 h of exposure, and from 1.44 to 22.00 μg/mL for *A. fischeri* [37,38,39]. These findings suggest that TER is less toxic than several phytochemicals commonly investigated for antimicrobial applications.

TER is a monoterpenoid alcohol that is partially soluble in water (2.54 g/L at 20 °C), a property that enhances its bioavailability. Its relatively low molecular weight (154.25 g/mol), weak acidic nature, and lack of charge at approximately neutral pH (Table S3) facilitate diffusion across biological membranes. Like other monoterpenes, TER interacts with phospholipidic bilayers, disrupting membrane integrity and increasing cellular permeability. This effect may ultimately lead to cell death, and could explain the relatively high toxicity observed in both bioindicators [11,40,41,42].

In *D. magna*, TER uptake may occur through direct contact with the body surface or through ingestion. Its relatively high log K_ow_ values (Table S3) favours interactions with biological surfaces, whereas the filter-feeding behaviour of cladocerans increases their exposure to dissolved contaminants, as described for other members of the order *Cladocera* [43]. In addition, the lipid-solubilising properties and moderate volatility of TER [44] may promote accumulation at the air-water interface, further enhancing uptake and toxicity.

The small molecular size and low molecular weight of TER may also facilitate its diffusion through outer membrane porins in *A. fischeri*. Once in the periplasmic space, TER may induce additional membrane disruption and cell damage, thereby enhancing its toxic effects [11]. In this context, Li et al. [45] reported similar alterations in *Escherichia coli*, including cytoplasmic leakage and cell lysis following exposure to TER.

All ABXs evaluated in this study were less toxic than TER in both *D. magna* and *A. fischeri* (Table 1, Figure 1), which is consistent with previous reports using the same bioindicators. For *D. magna*, published data indicated *EC*_50_ values of 875.5 µg/mL for GTM [46], 500 μg/mL for CHL [47], and values exceeding 100 μg/mL for both TC [48] and FF [49]. Similarly, previous studies on *A. fischeri* reported *EC*_50_ values of 537.5 µg/mL for CHL [50] and greater than 10,000 µg/mL for GTM [46]. The agreement between the present findings and the available literature supports the reliability of the toxicity data obtained in this study.

### 2.2. Toxicity of SACTAs in D. magna and A. fischeri

**Figure 2:**
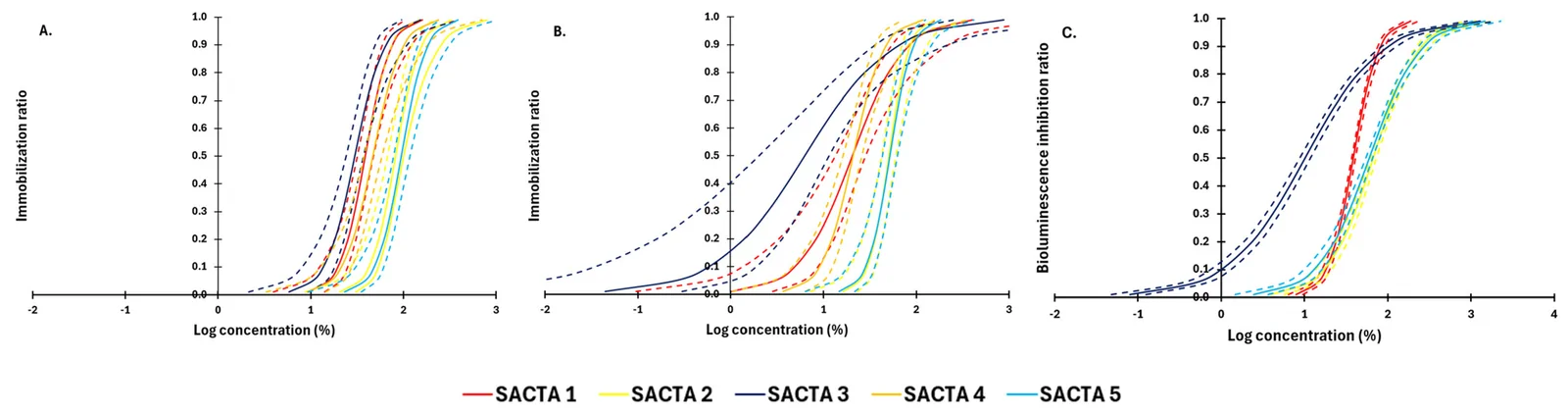
Dose–response curves, in percentages, for *Daphnia magna* after 24 h (**A**) and 48 h (**B**), and for *Allivibrio fischeri* after 30 min (**C**) of exposure to Synergistic Antimicrobial Combinations of terpineol and ABXs (SACTAs). Solid lines represent the fitted mean response derived from experimental data, while dashed lines indicate the 95% confidence intervals. Notably, for *A. fischeri*, SACTA 4 was excluded due to methodological incompatibility. The quantitative *EC*_50_ values and their 95% confidence intervals can be consulted in Table 2. Complete data on logit fitting are provided in Table S2.

Figure 2 presents the dose–response curves obtained for both bioindicators after fitting the experimental data to a logit regression model (Section 3.5.1).

To facilitate comparison among the different SACTAs, the proportions of the original compounds expressed as percentages were converted to concentrations (μg/mL) based on the initial formulation of each combination (Table 2). After 48 h of exposure, the toxicity ranking in *D. magna* was SACTA 3 > SACTA 1 ≈ SACTA 4 > SACTA 2 ≈ SACTA 5. Similarly, after 30 min of exposure, the toxicity ranking was SACTA 3 > SACTA 1 > SACTA 5 > SACTA 2 (Table 2, Figure 2).

Overall, the toxicity of SACTAs varied according to the ABX included in each formulation. Combinations containing CHL were consistently the most toxic to both bioindicators, which agrees with the relatively high toxicity observed for CHL when tested individually (Table 1 and Table 2). In contrast, formulations containing FF and GTM exhibited the lowest toxicity values. Notably, these combinations also contained lower proportions of TER, suggesting that the concentration of TER may play an important role in determining the overall toxic response of the mixtures.

A comparison between the *EC*_50_ values of the SACTAs (Table 2) and those of the corresponding ABXs (Table 1) revealed that the combinations were generally more toxic than the ABXs alone. However, the *EC*_50_ values for all SACTAs remained within the toxicity range observed for TER (Table 1), indicating that TER is likely the main contributor to the overall toxicity of these formulations.

Because the SACTAs were originally selected based on their enhanced antimicrobial activity, a corresponding increase in toxicity, particularly towards *A. fischeri*, might have been expected. However, the observed results revealed a more complex pattern of interactions.

#### 2.2.1. Synergistic Toxicity of SACTA 3 in *D. magna* and *A. fischeri*

**Figure 3:**
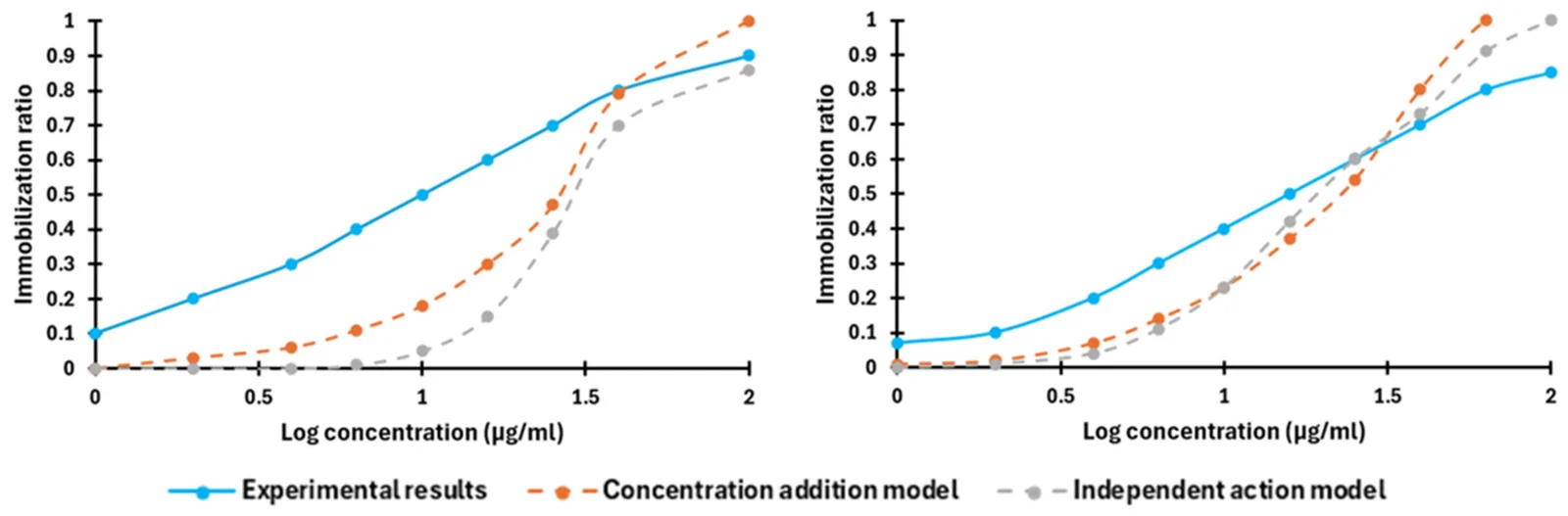
Graphical comparison of experimental toxicity responses and predicted values based on the concentration addition and independent action models for SACTA 3. Plots are shown for *D. magna* after 48 h of exposure (**left**) and *A. fischeri* after 30 min of exposure (**right**). The experimental dose–response curve is shown together with the two predictive curves derived from the CA and IA models. Further details on the toxicity models are provided in Section 3.5.1.

The only synergistic interaction observed in *D. magna* was associated with SACTA 3 (Figure 3), which is consistent with the fact that CHL exhibited the highest individual toxicity among the tested ABXs (Table 1). For this mixture, both the concentration addition (CA) and independent action (IA) models underestimated the experimentally observed toxicity, indicating that additional interaction mechanisms contributed to an enhanced toxic response (Figure 3). A similar synergistic effect was also detected in *A. fischeri*. However, at the highest tested concentrations, the experimental toxicity tended to decrease relative to the values predicted by both models (Figure 3).

These findings are consistent with previous studies reporting that CHL-containing mixtures are among the most toxic ABX combinations towards *D. magna* [51]. Enhanced toxicity following combined exposure to ABXs, pharmaceuticals, and phytochemicals has also been reported, even when the individual compounds were present at relatively low concentrations [52,53,54].

The elevated toxicity of SACTA 3 may be explained by the complementary modes of action of TER and CHL. The ABX can penetrate both the outer and cytoplasmic membranes of Gram-negative bacteria, and reach its ribosomal target, a process facilitated by its lipophilicity, water solubility, and uptake through membrane porins. Indeed, reduced porin expression or mutations affecting porin channels have been shown to decrease CHL permeability in bacteria such as *Haemophilus influenzae* and *Klebsiella* spp. [55,56].

Additional mechanisms may involve oxidative stress and disruption of cellular defence systems. In this context, CHL has been associated with the production of reactive oxygen species (ROS) in bacteria [57], whereas TER-induced membrane disruption may compromise resistance mechanisms such as efflux pumps and enzymatic detoxification pathways, including CHL-acetyltransferase activity [58,59]. The simultaneous action of these mechanisms could increase cellular susceptibility to the mixture, thereby reducing *EC*_50_ values and enhancing toxicity.

Although CHL primarily targets bacteria, its toxic effects have also been documented in metazoans such as *D. magna*. Exposure to CHL has been associated with oxidative stress, cellular damage, bioaccumulation, and reproductive impairment. Acute exposure may induce tissue damage, as well as oxidative and cellular stress responses, whereas chronic exposure has been linked to reduced fecundity and impaired development [47,51,60]. Therefore, the synergistic toxicity observed for SACTA 3 may result from the combination of TER-induced membrane disruption and CHL-mediated oxidative stress. Moreover, the filter-feeding behaviour of *D. magna* may facilitate the uptake of both compounds simultaneously, further increasing exposure and contributing to the overall toxic effect [61].

#### 2.2.2. Antagonistic Toxicity: SACTA 2 and SACTA 5 in *D. magna*

**Figure 4:**
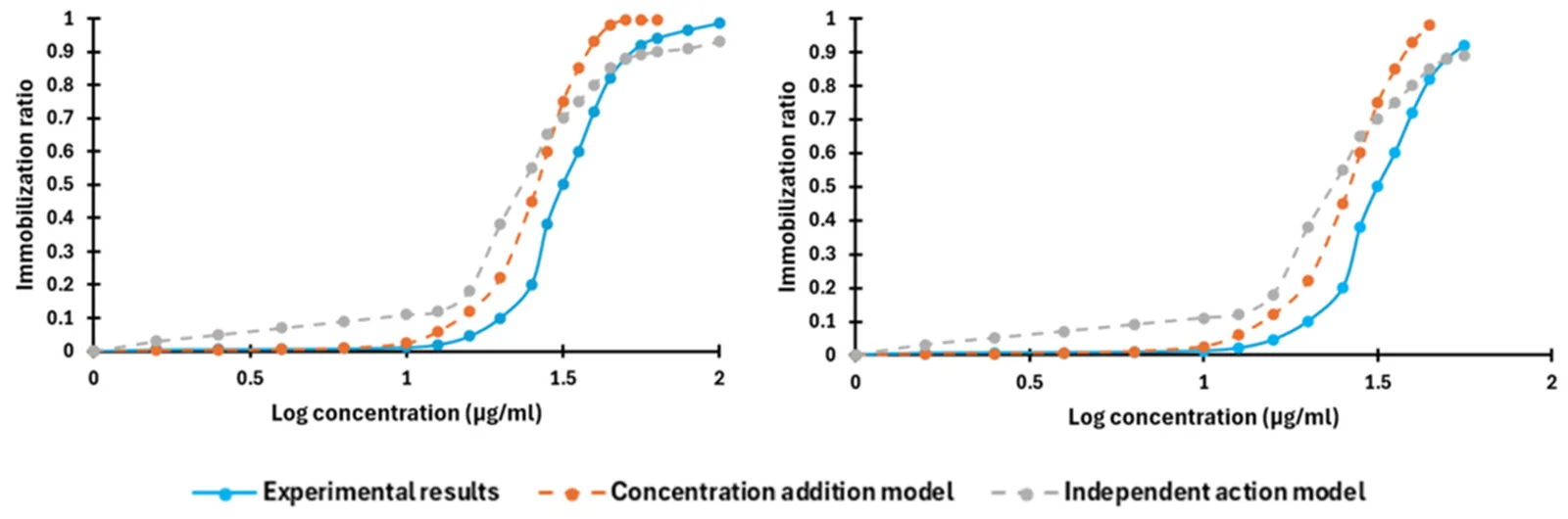
Graphical comparison of experimental toxicity responses and predicted values based on the concentration addition and independent action models for SACTA 2 (**left**) and SACTA 5 (**right**) for *D. magna* after 48 h of exposure. The experimental dose–response curve is shown together with the two predictive curves derived from the CA and IA models. Further details on the toxicity models are provided in Section 3.5.1.

Figure 4 shows the responses of *D. magna* exposed to SACTA 2 and SACTA 5, corresponding to TER combined with GTM and FF, respectively. In both cases, the CA and IA models predicted higher toxicity than that observed experimentally, indicating antagonistic interactions. Although CA- and IA-based curves could not be generated for *A. fischeri*, antagonistic effects may also be inferred for SACTAs 1, 2, and 5, as their *EC*_50_ values were higher than that observed for TER alone (Table 1 and Table 2).

The relatively low toxicity of GTM in *D. magna* may be partly explained by limited uptake and reduced interaction with biologically relevant targets, although the mechanisms underlying aminoglycoside toxicity in metazoans remain poorly understood [46]. In bacteria, aminoglycosides bind to the 30S ribosomal subunit, disrupting mRNA translation and leading to the production of aberrant proteins and the dissociation of polysomes into non-functional complexes [62]. GTM uptake is also influenced by membrane properties. In Gram-negative bacteria, GTM can destabilize the outer membrane by displacing lipopolysaccharide-associated Mg^2+^ and Ca^2+^ ions, thereby facilitating its own entry into the cell [63,64]. However, its relatively large molecular size and dependence on specific membrane interactions may limit its bioavailability in non-target organisms such as *D. magna*, which could contribute to the comparatively high *EC*_50_ values observed in this study (Table 1).

Similarly low acute toxicity has been reported in other biological models, including *A. fischeri* [46] and *Danio rerio* [65]. However, the response observed here in *D. magna* suggests that mechanisms beyond the canonical antibacterial mode of action may also be involved. Evidence from bacterial studies indicates that reduced membrane permeability, porin modifications, and biofilm-associated processes can decrease GTM uptake and toxicity [66,67]. Although these mechanisms cannot be directly extrapolated to crustaceans, they support the broader hypothesis that limited uptake and bioavailability play an important role in reducing GTM toxicity.

In contrast, FF has a lower molecular weight and moderate lipophilicity (Table S3), characteristics that may facilitate passive diffusion across biological membranes. In *D. magna*, these properties may enhance uptake through ingestion during filter-feeding activities. Previous studies have reported that FF exposure can induce oxidative stress, reproductive toxicity, alterations in gut microbiome, and potential mitochondrial and endocrine dysfunction [60,68,69]. Despite these effects, FF exhibited low toxicity toward *A. fischeri* when tested individually (Table 1), possibly as a consequence of resistance mechanisms such as enzymatic inactivation by acetyltransferases and increased efflux pump activity [70,71,72].

The antagonistic interactions observed for SACTAs 2 and 5 may result from several mechanisms, including reduced bioavailability and altered uptake dynamics. In the case of GTM, its physicochemical properties may promote interactions with TER [73], potentially reducing the availability of one or both compounds in the aqueous phase. In addition, exposure to GTM or FF may activate detoxification, biotransformation, or cellular defence pathways capable of modulating TER toxicity, thereby decreasing the overall toxic response while preserving parts of its membrane-disrupting activity. Similar antagonistic responses have been reported for pesticide mixtures in *D. magna*, where enzymatic inhibition or altered biotransformation pathways reduced the formation of toxic metabolites and consequently diminished mixture toxicity [52,74].

#### 2.2.3. Additive Toxicity: SACTA 1 and SACTA 4 in *D. magna*

**Figure 5:**
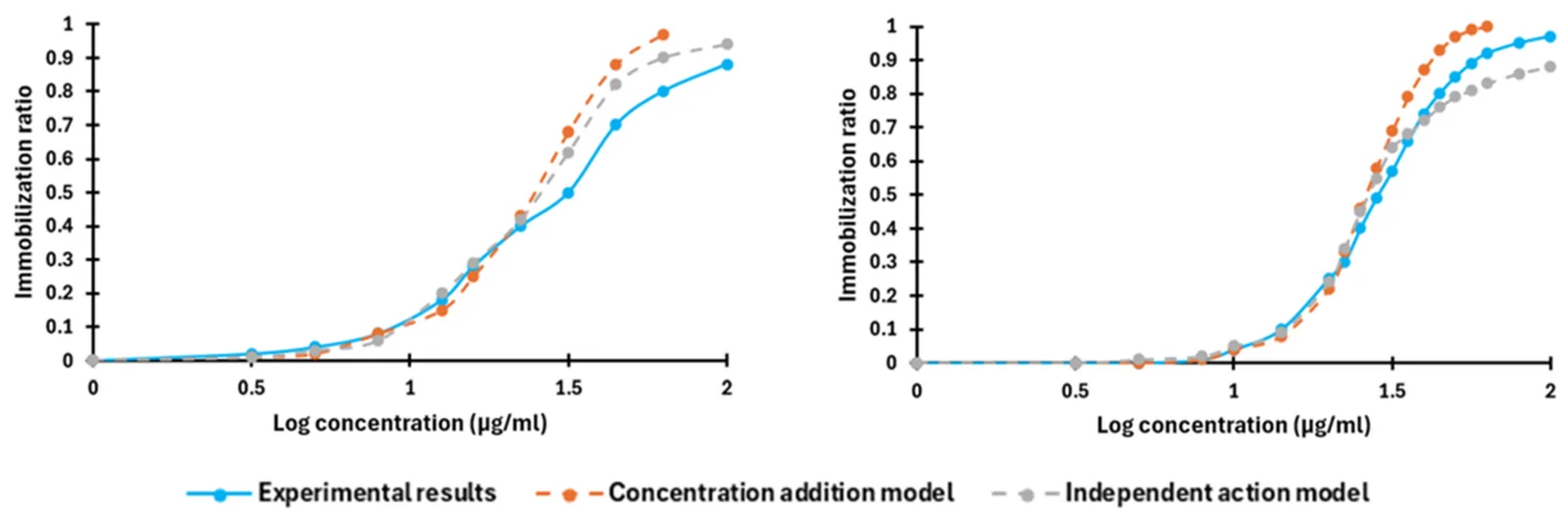
Graphical comparison of experimental toxicity responses and predicted values based on the concentration addition and independent action models for Synergistic Antimicrobial Combinations of terpineol and ABXs (SACTAs) 1 (**left**) and 4 (**right**) for *D. magna* after 48 h of exposure. The experimental dose–response curve is shown together with the two predictive curves derived from the CA and IA models. Further details on the toxicity models are provided in Section 3.5.1.

As shown in Figure 5, the toxicity predicted by the CA and IA models for SACTAs 1 and 4 closely matched the experimental data, indicating additive behaviour and suggesting that the combined effects were consistent with the sum of the individual components. Although the observed response for SACTA 1 was slightly lower than predicted, the 48 h *EC*_50_ confidence intervals overlapped with the predicted values (Table 2), indicating good agreements between the modelled and experimental datasets.

In the case of TC, its physicochemical properties may partly explain the toxicity observed in *D. magna*. Due to its relatively small molecular size (Table S3), amphipathic nature, and acid-base properties, TC can occur in neutral or partially ionized forms under physiological conditions, facilitating diffusion across biological membranes. Moreover, TC can form complexes with divalent cations such as Mg^2+^ and Ca^2+^, which may influence its transport across cellular barriers [75]. Uptake may also be mediated by eukaryotic membrane transporters, including organic anion transporters [76].

In *D. magna*, TC has been reported to bioaccumulate primarily through aqueous exposure rather than dietary intake, leading to alterations in gut microbiota communities and potentially disrupting physiological homeostasis [77]. Furthermore, multigenerational exposure to TC has been associated with stress-related transcriptional responses involving protein synthesis, carbohydrate metabolism, oxidative phosphorylation, and other key cellular processes [78].

Additive effects have also been reported for other mixtures of antimicrobial compounds. For example, mixtures of triclosan and triclocarban showed no significant differences between observed toxicity and model predictions [79]. Overall, the present findings support the view that antimicrobial mixtures may exhibit different interaction patterns, including additive, synergistic, and antagonistic effects, depending on the physicochemical properties and mechanisms of action of the individual components.

Similar complex interaction patterns have been described for mixtures containing organotin compounds [80], ZnO and CuO nanoparticles [81], and various classes of pesticides [52]. These observations reinforce the importance of evaluating each antimicrobial formulation individually, as mixture toxicity cannot always be predicted from the effects of the individual compounds alone.

### 2.3. Impact of SACTAs on a Riverine Microbiome

#### 2.3.1. Microbiome Characterisation via 16S rRNA Gene Metabarcoding

**Figure 6:**
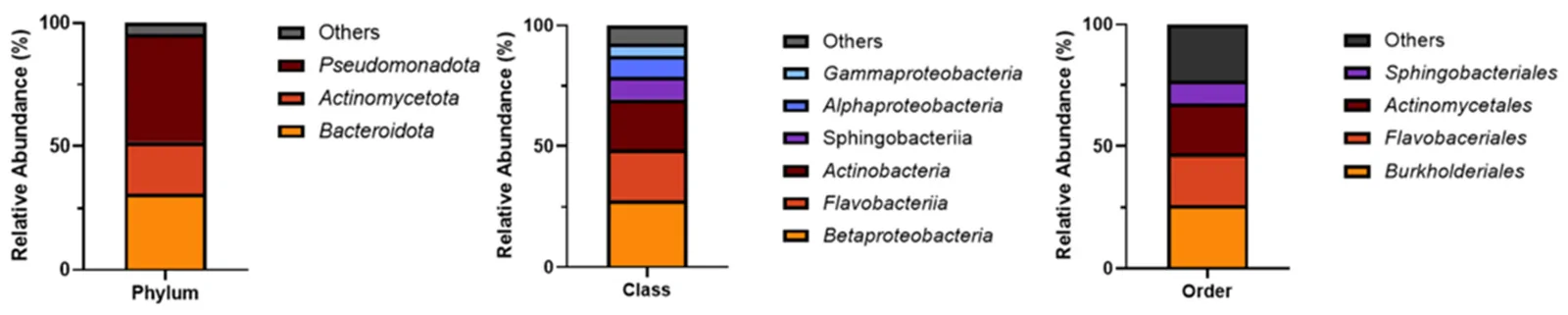
Characterisation of Gállego river microbiome using 16S rRNA V3-4 region metabarcoding analysis. Stacked bars show the relative abundance of operational taxonomic units (OTUs) at the phylum (**left**), class (**centre**) and order (**right**) taxonomic levels. In all cases, the “Others” category represents the sum of OTUs with relative abundances below 3.50%. A complete report on the outcomes of the sequencing assay is provided in Figure S1.

As shown in Figure 6, the riverine microbiome collected from the Gállego River (Zaragoza, Spain) was preliminarily characterized using 16S rRNA gene metabarcoding. This analysis provides ecological context for interpreting the subsequent evaluation of SACTAs effects on the metabolic fitness of the microbial community. Microorganisms are essential components of freshwater ecosystems, where they contribute to key biogeochemical processes, including organic matter decomposition, nutrient cycling, and greenhouse gas regulation. Consequently, alterations in microbial community structure may have broader implications for ecosystem functioning and stability [82].

Taxonomic classification yielded high identification rates, with more than 90% of the detected taxa being assigned at the genus level. However, only 24.07% of the sequences could be identified at the species level (Figure S1). The microbial composition was consistent with that typically reported for freshwater ecosystems [83], being dominated by the phyla *Pseudomonadota*, *Bacteroidota*, and *Actinomycetota*. This distribution indicates a clear dominance of Gram-negative bacteria within the community (Figure 6). In addition, 4.52% of the bacterial sequences remained unclassified, highlighting the high taxonomic diversity commonly found in riverine microbiomes (Figure S1).

These dominant phyla encompass a broad range of metabolic strategies, including aerobic, facultative anaerobic, and anaerobic chemoorganotrophic, as well as nitrogen-fixing microorganisms [84,85,86].

Among them, *Pseudomonadota* was the most abundant phylum, consistent with previous studies reporting that members of this group may account for approximately 40% of bacterial populations in freshwater environments [87]. Owing to their rapid growth rates and metabolic versatility, these bacteria are often among the first responders to fluctuations in nutrient availability.

As commonly reported in freshwater systems, *Betaproteobacteria* and *Alphaproteobacteria* were the predominant classes with *Pseudomonadota*, followed by *Gammaproteobacteria* (Figure 6) [88]. Most *Betaproteobacteria* were affiliated with the order *Burkholderiales*, in agreement with previous observations in river ecosystems [88,89,90]. Members of this group are frequently associated with nutrient cycling and organic matter transformation in aquatic environments.

The *Alphaproteobacteria* community was mainly represented by *Rhodobacterales* and *Sphingomonadales*. These groups are often associated with oligotrophic or nutrient-limited environments and have been previously identified as abundant components of microbial communities in Spanish rivers [83,91]. Within *Gammaproteobacteria*, *Enterobacterales* was the most abundant order. Members of this taxon display considerable metabolic flexibility and are able to exploit a wide variety of carbon and nutrient sources [92].

The phylum *Bacteroidota* was also represented, supporting their recognised role in the degradation of organic matter in freshwater ecosystems (Figure 6). Within this phylum, *Flavobacteriia* and *Sphingobacteria*, were the dominant classes. These microorganisms are known to participate in the breakdown of high-molecular-weight organic compounds, including carbohydrates and proteins, thereby contributing to nutrient recycling in aquatic habitats [93]. These bacteria are also frequently detected in urban and anthropogenically influenced river systems [94,95].

Finally, *Actinomycetota*, represented predominantly by the order *Actinomycetales*, constitute another important component of the microbial community. Members of this group contribute significantly to organic matter turnover and to nitrogen and carbon cycling in freshwater ecosystems (Figure 6) [96]. In addition, their presence may reflect a high degree of ecological adaptability, as *Actinomycetales* are able to colonise environments affected by various pollutants, including heavy metals and organic pollutants [97].

#### 2.3.2. Community Metabolic Fluctuations Induced by SACTAs

In this part of the study, the Biolog EcoPlates^TM^ assay was used to evaluate changes at the community-level physiological profile (CLPP) of the microbial community following exposure to SACTAs. This approach was adopted to obtain a more environmentally relevant assessment of the potential ecological impact of these binary mixtures on freshwater ecosystems.

**Figure 7:**
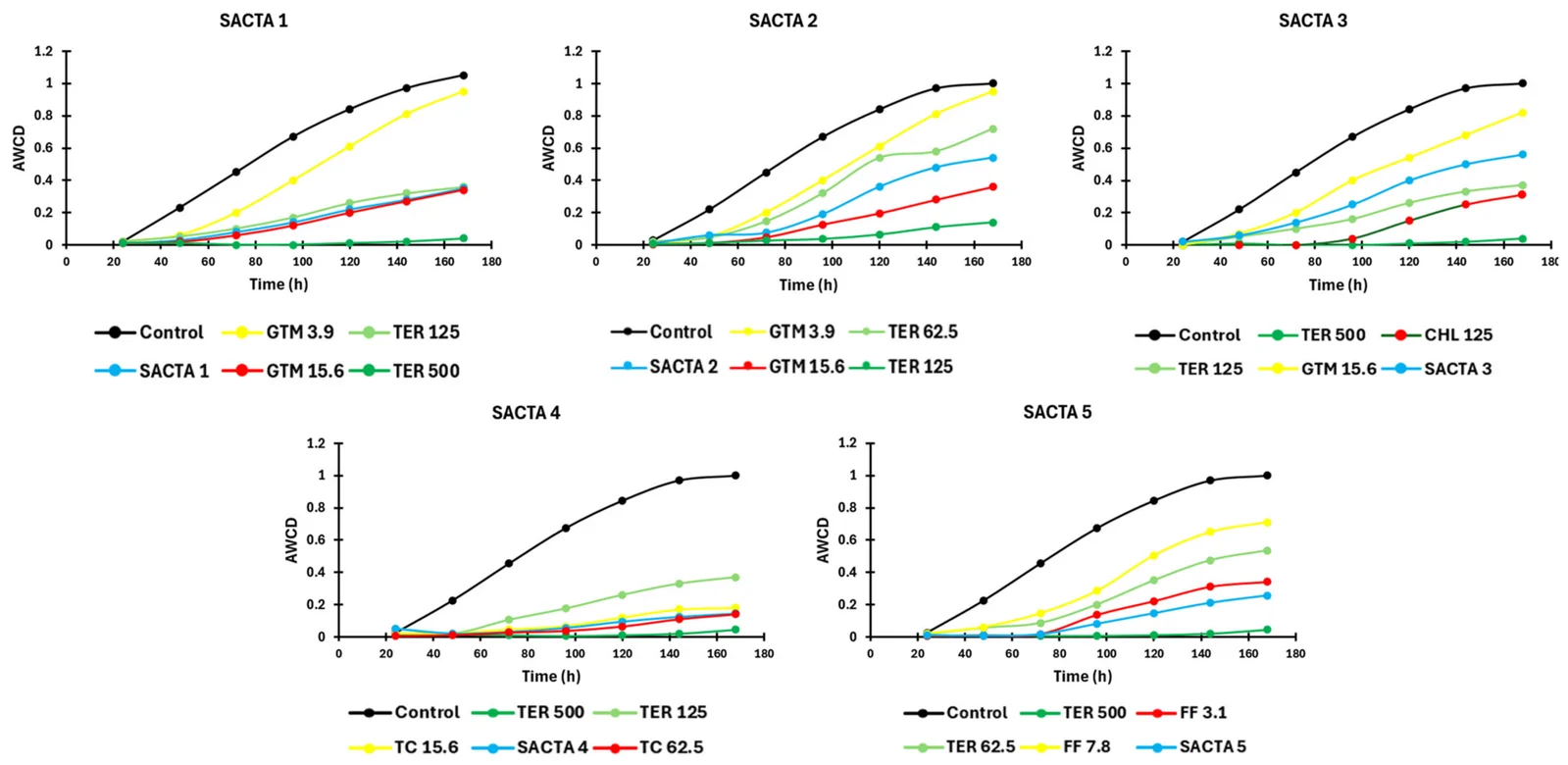
Biolog EcoPlates™ assays showing the riverine microbiome average well colour development (*AWCD*) following SACTA exposure. Each line represents the values (in μg/mL) regarding the control (black), TER at its minimum inhibitory concentration (MIC) value (green), ABX at MIC concentration (red), TER at SACTA concentration (light green), ABX at SACTA concentration (yellow) and binary SACTA (blue) obtained by fitting plate absorbances to Equation (5) (see Section 3.5.1.). Further details regarding the different SACTAs concentrations are provided in Table 4.

**Figure 8:**
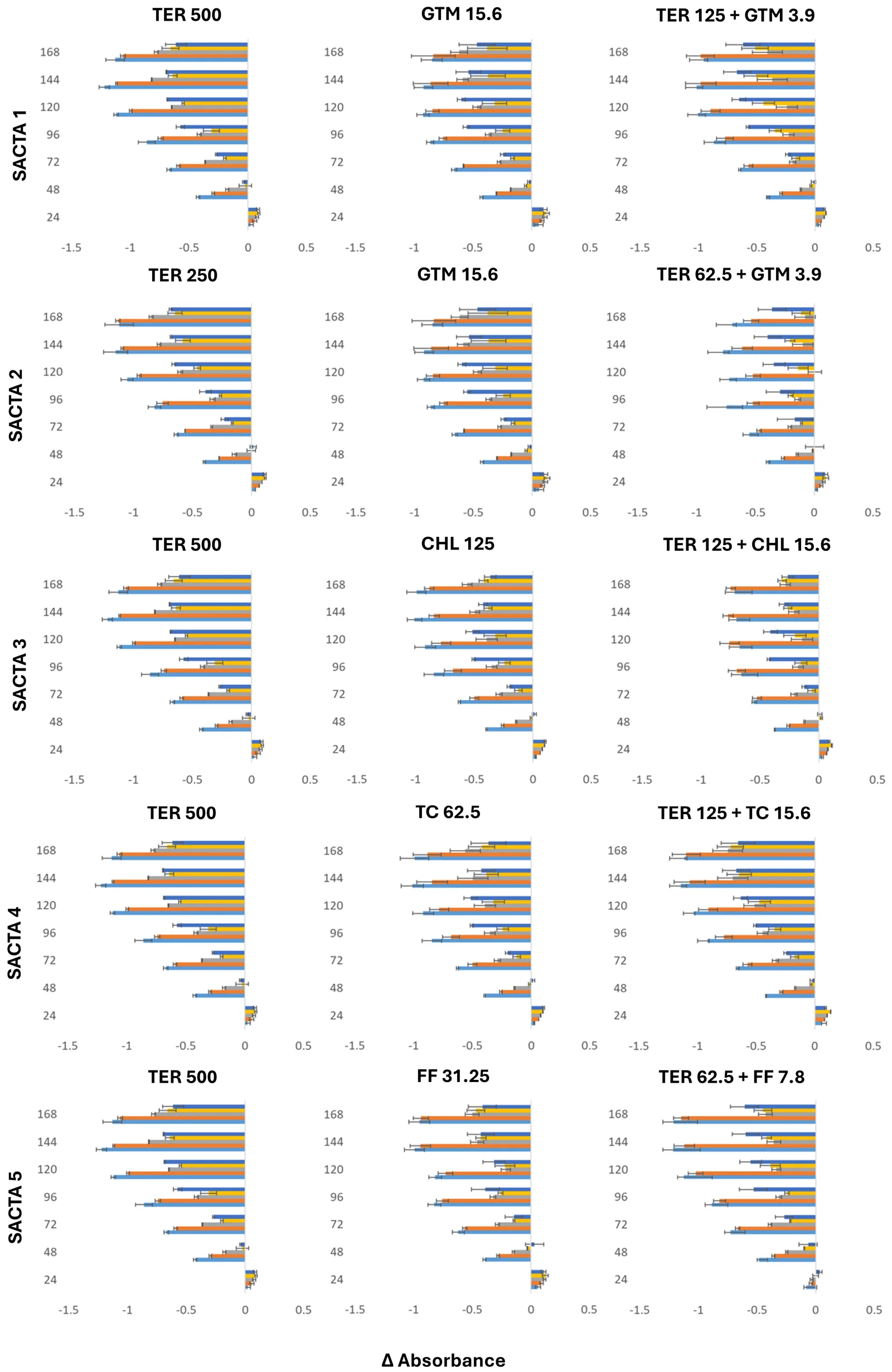
Biolog EcoPlates ™ assays showing the riverine microbiome community-level physiological profile (CLPP) following SACTA exposure. Results are expressed as the difference in absorbance (*AWCD*) regarding the control and categorised by metabolite groups. The left and central columns represent the effects at the minimum inhibitory concentration (MIC) of TER and the corresponding ABX, respectively, while the right column shows the effects of the binary treatment. In each plot, the concentration of the compound(s) in μg/mL is indicated.

The effects of the five SACTAs on the overall metabolic fingerprint of a natural riverine microbial community, expressed as average well colour development (*AWCD*), are shown in Figure 7. To gain further insights into the metabolic responses induced by SACTAs, the data were additionally analysed according to the five substrate groups represented in the Ecoplates, namely polymers, carbohydrates, carboxylic and ketonic acids, amino acids, and amines/amides (Figure 8). Complete metabolic profiles for all SACTAs are provided in Figures S2–S6.

The results indicate that all five SACTAs exerted lower or comparable ecotoxicological effect on community metabolism than the corresponding ABXs tested alone at their MIC values (Table 3). In contrast, TER at its MIC, produced the greatest inhibitory effect on microbial metabolic activity (Table 3). Notably, the toxicity pattern observed for the microbial community differed substantially from that obtained using the individual bioindicators (Table 2). Based on the reduction in *C_max_* (maximum attainable AWCD value, used as an estimate of the carrying capacity or maximum metabolic activity of the microbial community) relative to the control (Table 3), the toxicity ranking of the SACTAs was as follows: SACTA 5 > SACTA 1 > SACTA 4 > SACTA 3 > SACTA 2.

Accordingly, SACTA 5, which contains FF, was the most toxic combination towards the riverine microbiome. This finding contrasts with the results obtained for *D. magna* and *A. fischeri*, in which the CHL-containing combinations (SACTA 3), exhibited the highest toxicity (Table 2).

When the effects of SACTAs were compared with those of TER and the corresponding ABX tested individually at their synergistic concentrations, the interactions were predominantly antagonistic or additive, as discussed below. No evidence of synergistic toxicity was observed at the microbial community level. This contrasts with the responses recorded in the individual bioindicators and highlights the importance of integrating *CLPP* analyses with conventional ecotoxicological assays to achieve a more comprehensive and environmentally realistic evaluation of phytochemical–ABX mixtures.

To the best of our knowledge, this is the first study to assess the combined effects of TER with synthetic ABXs on the metabolism of a natural freshwater microbiome. Nevertheless, previous studies investigating commercial ABX mixtures in sediment-associated microbial communities have reported discrepancies between responses observed in individual test organisms and those detected in complex environmental microbiomes [98]. Furthermore, several studies evaluating commercial ABX mixtures have described synergistic effects on aquatic microorganisms [99,100,101,102], in contrast to the predominantly antagonistic and additive responses observed for the SACTAs investigated here.

#### 2.3.3. SACTAs with Antagonistic Toxicity Effects

For SACTAs 2, 3, and 4, containing GTM, CHL and TC, respectively, *C_max_* values decreased by 16.52–41.56% relative to the control (Table 3). However, these reductions were smaller than those predicted from the combined effects of TER and the corresponding ABX tested individually at their synergistic concentrations (Table 3) indicating an antagonistic interaction. In other words, the observed toxicity of these mixtures was lower than the toxicity expected from the sum of the effects of their individual components. Although antagonistic interactions among commercial ABXs are relatively uncommon, antagonism has been reported in the context of antimicrobial resistance development [103].

TER alone exhibited higher toxicity than the corresponding SACTAs, both at its MIC and at the synergistic concentration, whereas the ABXs generally produced marked toxic effects only when tested at their MICs. Previous studies have shown that CHL and TC can substantially affect riverine microbial communities by reducing growth and altering metabolic activity when applied individually [36]. Moreover, TC has been reported to impair sludge integrity in wastewater treatment systems, by promoting floc disintegration and cell rupture [104].

Interestingly, the toxicity of these SACTAs was similar to, or even lower than, that of the corresponding ABX tested alone at its MIC, particularly in the case of GTM and CHL (Table 3). This observation suggests that selected TER–ABX combinations may contribute to reducing ABX consumption while limiting the ecological risks associated with ABX residues released into aquatic environments.

Analysis of the metabolic profiles revealed that polymer and carbohydrate utilization were the most strongly affected functional categories, showing marked reductions across treatments (Figure 8). The greatest decreases were observed for GTM and TER when tested at their MICs, whereas the effects associated with the SACTAs were less pronounced, which is consistent with the antagonistic responses observed for microbial growth (Figure 7). This pattern may reflect the complexity of the riverine microbial community, which comprises microorganisms with different sensitivities to TER and ABXs, resulting in responses that are less predictable than those observed in single-species systems [105]. The coexistence of Gram-negative bacteria, mainly belonging to the phyla *Pseudomonadota* and *Bacteroidota*, together with Gram-positive members of *Actinomycetota*, introduces substantial variation in membrane structure, permeability, metabolic capacity, and tolerance to chemical stressors. Such taxonomic and functional diversity may promote compensatory mechanisms within the microbial community, including partial detoxification processes, reduced uptake of toxic compounds, or the replacement of sensitive taxa by more tolerant populations.

Furthermore, because TER and ABXs act through different mechanisms, their combined effects may alter community homeostasis without necessarily generating synergistic toxicity. Although TER–ABX mixtures reduced microbial growth, they did not completely suppress metabolic activity, except for SACTA 4 (Figure 7 and Figure 8). This finding suggests the presence of redundant metabolic functions and a broad diversity of biochemical pathways within the community, allowing partial functional compensation following exposure. Similar alterations in microbial community structure and function have been reported following exposure to FF [106]. In addition, environmental microbial communities possess a considerable capacity to develop tolerance to ABXs through a variety of physiological and genetic mechanisms [107,108,109]. Such adaptative responses may further contribute to the antagonistic or non-synergistic effects observed for these TER-ABX combinations.

#### 2.3.4. SACTAs with Additive Toxicity Effects

The results of this study suggest that TER–ABX combinations may represent a promising strategy for reducing ABX use while maintaining antimicrobial efficacy. In riverine microbial communities, the toxicity of SACTAs was comparable to, or lower than, that of the corresponding ABX tested at its MIC, indicating a potentially lower environmental burden than conventional ABX monotherapy (Figure 7, Table 3).

In contrast, toxicity assays performed with *D. magna* and *A. fischeri* showed that SACTAs were generally more toxic than the corresponding ABXs when *EC*_50_ values were expressed in µg/mL (Table 1 and Table 2). However, when toxicity was evaluated relative to the MIC of each ABX, SACTAs exhibited toxicity levels similar to those of FF, lower than those of GTM, and higher only for combinations containing CHL and TC. Furthermore, these bioindicators exhibited antagonistic, additive, and synergistic interaction patterns, indicating that the toxicity of TER-ABX mixtures depends both on the biological model used and on the specific composition of each SACTA.

By contrast, the CLPP analysis of the riverine microbial community revealed only additive or antagonistic responses, with no evidence of synergistic toxicity (Figure 7 and Figure 8). These findings highlight the importance of combining traditional single-species bioassays with community-level approaches to obtain a more comprehensive and environmentally relevant assessment of the ecotoxicological effects of phytochemical–ABX mixtures.

Although TER was generally more toxic than the ABXs evaluated in this study, the concentrations reported in wastewater effluents are substantially lower than the *EC*_50_ values determined here, suggesting a limited risk of acute toxicity under environmentally relevant conditions [18]. Moreover, incorporation of TER into SACTAs reduced its concentration to only 12.5–25% of its MIC. TER also appeared to be less persistent in aquatic environments than the ABXs investigated. For example, TC has been reported to persist in river water for more than 96 h [110], whereas FF and CHL remain relatively stable under low-oxidation conditions [111,112,113]. In contrast, TER undergoes rapid aerobic biodegradation [114], is considered readily biodegradable according to OECD TG 310 criteria [115], and its broad membrane-disrupting mode of action may reduce the selection pressure for specific resistance mechanisms [116].

Although the *EC*_50_ values of the tested ABXs generally exceeded their MICs, prolonged exposure to low concentrations may still affect aquatic microbial communities. Adverse effects have previously been reported for CHL and FF, including alterations in microbial community structure and function [117]. In addition, FF has been shown to induce gut dysbiosis and disrupt liver metabolism in fish [118]. The environmental risks associated with ABX may be further amplified when multiple compounds are present simultaneously, as additive or synergistic interactions have been described for several ABX mixtures, particularly CHL-containing combinations [119].

Overall, the SACTAs evaluated in this study reduced ABX doses by approximately 75% while maintaining antimicrobial efficacy. Because FF, TC, CHL and GTM have all been associated with the occurrence and dissemination of antimicrobial-resistant bacteria and resistance determinants in aquatic ecosystems [106,108,120,121,122,123,124,125], reducing ABX concentrations to approximately 25% of their original MIC values could represent a more environmentally sustainable alternative to conventional ABX monotherapy. Nevertheless, the ecological safety of these formulations remains dependent on their species-specific toxicity and environmental fate, highlighting the need for further assessment under environmentally relevant conditions.

## 3. Materials and Methods

### 3.1. Reagents

*α*-terpineol (TER) and tetracycline (TC) were purchased from Vidrafoc (Barcelona, Spain). Gentamicin (GTM), and chloramphenicol (CHL) were obtained from Acofarma (Madrid, Spain), while florfenicol (FF) was purchased from Laboratorios Karizoo, S.A. (Barcelona, Spain). All reagents were of analytical grade with purities ≥97%. The physicochemical properties of all reagents are provided in Table S3.

The concentrations of the SACTA stock solutions used in this study are presented in Table 4. To ensure suitable experimental conditions, the pH of each stock solution was measured using a Basic 20 pH meter (Vidrafoc, Barcelona, Spain). All solutions were freshly prepared on the day of each experiment. The pH was maintained within the range of 6.5–7.5 and, when required, adjusted using 0.1 M NaOH.

### 3.2. SACTAs Acute Toxicity in Daphnia magna

The effects of TER, ABXs, and SACTAs on *D. magna* were evaluated following the standardized protocol provided in the Daphtoxkit F^TM^ magna (Ref. DM121219, Vidrafoc, Barcelona, Spain), and in accordance with OECD guidelines [126]. To preserve reagent stability, the kit was stored in the dark at 4 ± 1 °C until use. Only neonates less than 24 h old, obtained from ephippia hatched for 72 h, were used.

*D. magna* ephippia were initially incubated for 72 h at 22 ± 0.5 °C under continuous illumination (6000 lx) using a TOXKIT incubator model CH-0120D-AC/DC (ECOTEST, Valencia, Spain). After hatching, neonates were fed with one vial of *Spirulina* supplied with the kit, and allowed to feed for 2 h before exposure.

Test solutions were freshly prepared for each assay by diluting stock solutions in synthetic freshwater prepared according to ISO 6341 [127]. Throughout the experiments, pH values were monitored and remained between 6.5 and 7.5, without requiring adjustment. Synthetic freshwater alone served as the negative control.

Each treatment was tested in five replicates, each containing five viable neonates. Neonates were exposed to TER at concentrations 20, 40, 60, 80, and 120 µg/mL in the presence of 3% DMSO. In addition, organisms were exposed to different fractions of the original SACTA concentrations (Table 4). For SACTAs 1, 3, and 4, exposure levels corresponded to 1×, 0.5×, 0.25×, 0.125×, and 0.00125× of the original concentration. For SACTAs 2 and 5, the tested levels were 2×, 1×, 0.5×, 0.25×, and 0.0025×. Exposed daphnids were incubated in the dark at 22 ± 0.5 °C for 24 h.

Following exposure, immobilisation was assessed. Individuals that failed to exhibit movement within 15 s after gentle agitation were considered immobile. These data were recorded and used to determine the median lethal concentration (LC_50_), as described below.

### 3.3. Bioluminescence Inhibition Assay in Aliivibrio fischeri

The acute toxicity of TER and SACTAs towards *A. fischeri* NRRL-B-11177 (Ref. 945006, Macherey-Nagel, Düren, Germany) was assessed using the bioluminescence inhibition assay, according to ISO 11348-3 [128]. Lyophilized bacteria were reconstituted using the supplied reactivation solution and maintained at 5 ± 1 °C for 5 min prior to testing to ensure culture viability.

All test solutions were prepared by diluting stock solutions in 2% NaCl. No pH adjustment was required. TER was evaluated at concentrations of 200, 20, 2, 0.2 and 0.02 µg/mL, in the presence of 3% DMSO. SACTA concentrations were prepared as fractions of their original concentrations. For SACTAs 1, 3, and 4, the tested ratios were 1×, 0.5×, 0.25×, 0.125× and 0.00125×. For SACTAs 2 and 5, the tested ratios were 2×, 1×, 0.5×, 0.025× and 0.0025×.

Assays were performed at 15 ± 1 °C. Briefly, 0.5 mL of bacterial suspension was dispensed into each test vial, and baseline bioluminescence was measured in triplicate using a Biofix^®^ Lumi-10 luminometer (Macherey-Nagel, Dueren, Germany). Subsequently, 0.5 mL of each test solution was added to the corresponding vial and incubated for 30 min. After exposure, bioluminescence was measured again using the same procedure. The resulting data were used to calculate the median effective concentration (*EC*_50_), as described below.

### 3.4. Impact of SACTAs on the Global Metabolic Activity of an Indigenous River Microbiome

#### 3.4.1. Sample Collection and Preprocessing

Water samples were collected from the Gállego River (Zaragoza, Spain) in May 2024. Sampling and transport to the laboratory were carried out according to ISO 19458 [129] using sterile containers.

Several physicochemical parameters were measured in situ. Water temperature was 17 °C, measured using a Nahita precision thermometer (ICT, S.L., La Rioja, Spain). The pH was 7.5, determined using a PanReac AppliChem A011435 pH meter (Darmstadt, Germany), while electrical conductivity was 2.8 mS/cm, measured using a Hanna HI8733 conductivity meter (Merck, Madrid, Spain).

On the day of collection, a 2 L aliquot was sent to Laboratorios Valero Analítica (Zaragoza, Spain) for complete physicochemical characterization. The results are presented in Table S4.

Sample preprocessing differed depending on the subsequent analysis. For high-throughput sequencing (HTS), 5 L of river water were vacuum-filtered through a 0.22 µm nitrocellulose membrane (Sartorius, Göttingen, Germany). Filters were resuspended in 50 mL sterile phosphate-buffered saline (PBS), and the resulting suspension was centrifuged at 5000× *g* for 10 min. The supernatant was discarded and the pellet stored at −80 °C until DNA extraction.

For ecotoxicological analyses, a 1 L water aliquot was filtered through a 70 µm nylon mesh (Becton Dickinson, Madrid, Spain) to remove coarse particulate matter. The filtered sample was subsequently stored at 4 °C in the dark until used.

#### 3.4.2. Microbiome Characterization by 16S rRNA Gene High-Throughout Sequencing

Total genomic DNA was extracted from the pre-processed samples using the AllPrep^®^ PowerViral^®^ DNA/RNA Kit (Qiagen, Barcelona, Spain), according to the manufacturer’s instructions. DNA concentration was quantified using PicoGreen^®^ (Thermo Fisher Scientific Inc, Walthan, MA, USA) fluorometric assays.

Amplification targeted the V3–V4 region of the 16S rRNA gene, using 1.5 ng of DNA per sample. The first PCR included an initial denaturation at 98 °C for 30 s followed by 21 cycles of denaturation at 98 °C for 10 s, annealing at 55 °C for 20 s and extension at 72 °C for 20 s, and a final extension step at 72 °C for 2 min. It was performed using Q5^®^ Hot Start High-Fidelity DNA Polymerase (New England Biolabs, Ipswich, MA, USA) and 100 nM specific primers. Successful amplification was verified by agarose gel electrophoresis.

The second PCR (indexing) started with an initial denaturation at 98 °C for 30 s, followed by 13 cycles of 98 °C for 10 s, 55 °C for 20 s and 72 °C for 20 s, and a final extension at 72 °C for 2 min. It was performed using 400 nM barcoded primers from the Access Array Barcode Library for Illumina Sequencers (Fluidigm, South San Francisco, CA, USA), allowing sample indexing and library completion.

Library size distribution and concentration were assessed using an Agilent TapeStation system. Libraries were pooled at equimolar concentrations, purified with AMPure beads, and quantified by qPCR using the KAPA SYBR^®^ FAST qPCR kit for LightCycler^®^ 480 (Sigma-Aldrich, Madrid, Spain).

The pooled library was denatured and loaded onto the sequencing flow cell at a final concentration of 10 pM. Sequencing was performed using a MiSeq Reagent Kit v3 (2 × 300 bp paired-end reads) on an Illumina MiSeq platform.

Raw sequence data were converted into FASTQ files using Illumina bcl2fastq software (v2.20.0.422 (San Diego, CA, USA)). Phylogenetic and taxonomic analyses were conducted using the 16S Metagenomics workflow available in BaseSpace v1.1.0 (Illumina, Madrid, Spain), employing the Greengenes v13.5 database [130] for operational taxonomic unit (OTU) assignment.

#### 3.4.3. Community-Level Physiological Profiling (CLPP)

The effects of TER and SACTAs on the metabolic activity of the riverine microbiome were assessed using Biolog EcoPlates^TM^ assays (Tiselab, Barcelona, Spain). This approach evaluates the utilisation of 31 carbon sources in triplicate and has been widely applied to characterize community-level metabolic responses [33,131].

TER, individual ABXs at synergistic and MIC concentrations, and SACTAs (Table 1) were freshly prepared in sterile deionised water. TER-containing solutions included 1.25% DMSO. The pH of all solutions was adjusted to 6.5 ± 0.5.

Each Ecoplate was assigned to a single treatment. Briefly, 100 µL of the corresponding test solution was added to each well, followed by 100 µL of the preprocessed river water used as microbial inoculum.

Immediately after plate preparation, optical density (OD) at 590 nm was measured using a Synergy H1 microplate reader (BioTek, Dallas, TX, USA) equipped with Gen5™ v2.0 software. Plates were then incubated under static conditions in the dark at 25 ± 0.5 °C. OD measurements were subsequently recorded every 24 h for a total incubation period of 168 h.

Carbon utilisation was quantified based on the reduction of tetrazolium violet dye, following the methodology described by Pohland and Owen [132].

### 3.5. Data Processing and Statistical Analysis

#### 3.5.1. Dose–Response Modelling for *D. magna* and *A. fischeri*

Median lethal concentration (LC_50_) and median effective concentration (*EC*_50_), defined as the concentration producing 50% of the maximum response, were calculated for *D. magna* immobilization and *A. fischeri* bioluminescence inhibition, respectively.

Dose–response data were fitted using a logit model as previously described [133], applying maximum likelihood estimation with XLSTAT v2014.5.03 (Lumivero/Addinsoft, Long Island City, NY, USA) for Microsoft Excel (Microsoft 365 Apps for enterprise):

(1) $$R=\left(\frac{1}{1+e^{\left[-\left[\beta_{0}+\beta_{1}D\right]\right]}}\right)$$

where *R* represents the response at dose *D*; *D* is the log_10_ transformed of the tested concentration (µg/mL); *β*_0_ is an empirical parameter; and *β_1_* is the specific response coefficient.

*EC*_50_ values were subsequently calculated using Equation (2):

(2) $$EC_{50}=-\frac{\beta_{0}}{\beta_{1}}$$

Model performance was evaluated using the likelihood ratio statistic (*LR*(χ^2^)), with statistical significance defined at a confidence level of 95% or higher.

To assess mixture toxicity, experimental responses were compared with the predicted responses of two theoretical reference models: concentration addition (CA) and independent action (IA), which assume similar or dissimilar modes of action, respectively [134].

The CA model was applied according to Loewe and Muischnek [135] (Equation (3)):

(3) $${EC}_{pCA}=\left(\sum_{i}\frac{C_{i}}{C_{pi}}\right){EC}_{p}$$

where *EC_pCA_* is the concentration predicted to produce a response proportion *p* under the CA assumption; *C_i_* is the concentration of component *i* within the mixture; and *C_pi_* is the concentration of the same component producing the response proportion *p* when tested individually.

The IA model was calculated as follows:

(4) $${EC}_{pIA}=\left[1-\prod_{i}\left(1-C_{i}{EC}_{i}\right)\right]/C_{mix}$$

where *EC_pIA_* is the predicted concentration producing a response proportion *p* under the IA model; *EC_i_* corresponds to the effect produced by the component *i* at concentration *C_i_*; and *C_mix_* is the total concentration of all compounds in the mixture.

Experimental and predicted responses were subsequently compared graphically. Interactions were classified as synergistic when observed effects exceeded model predictions, antagonistic when observed effects were lower than predicted, and additive when experimental and theoretical responses closely matched.

#### 3.5.2. Processing of River Microbiome CLPP Data

Average well color development (*AWCD*) was calculated for each sampling time using Biolog Ecoplates™ data, according to Weber et al. [136] (Equation (5)):

(5) $$AWCD=\sum_{i=0}^{7}\left({OD}_{t=xi}-{OD}_{t=x0}\right)$$

where OD_*t*=*xi*_ represents the optical density measured at a given sampling time and OD_*t*=*x*0_ corresponds to the initial measurement. Negative *AWCD* values were set to zero.

*AWCD* data were then fitted to Equation (6) using a least-squares approach (quasi-Newton algorithm) implemented in the SOLVER module of Microsoft Excel 365:

(6) $$AWCD=\frac{C_{max}}{1+e^{\;b-rt}}$$

where *C_max_* represents the maximum attainable population size; *r* in the intrinsic growth rate (h^−1^); *b* is an empirical parameter, and *Tm*_50_ is the time required to reach 50% of the carrying capacity.

Carbon substrates were grouped according to Peleg et al. [137] to evaluate microbial substrate utilization patterns. *AWCD* values for each substrate category were calculated using Equation (5) and expressed as the mean of three independent replicates.

Finally, *EC*_50_ values expressed as percentages of the total SACTA concentration. for *A. fischeri* (30 min) and *D. magna* (48 h) were adjusted using Holm–Šídák’s multiple-comparison procedure.

## 4. Conclusions

This study evaluated the ecotoxicity of five SACTA formulations and their individual components in freshwater bioindicators and riverine microbial communities, considering their previously demonstrated antimicrobial efficacy and potential for reducing ABX dosage. The results revealed complex toxicity patterns that varied according to the ABX included in each formulation.

In *D. magna* and *A. fischeri*, CHL was the most toxic ABX, both when tested individually and when incorporated into SACTA formulations. Overall, SACTAs exhibited lower *EC*_50_ values than the corresponding ABXs tested alone, indicating higher toxicity. However, when compared with the toxicity associated with ABX MIC values, increased toxicity was observed only for formulations containing CHL and TC (SACTA 3: *EC*_50_ = 0.98 µg/mL in *D. magna* and 1.59 µg/mL in *A. fischeri*; SACTA 4: *EC*_50_ = 3.25 µg/mL in *D. magna*). For the remaining combinations, toxicity was like or lower than that of the corresponding ABXs at their MICs.

In contrast, riverine microbial communities displayed markedly different responses patterns. The FF-containing SACTA exhibited the highest toxicity (SACTA 5: *C*max = 0.26 vs. 1.06 in control; 75.15% inhibition), while additive and antagonistic interactions predominated over synergistic effects. Furthermore, all SACTAs showed toxicity levels comparable to or lower than those of the corresponding ABXs tested at their MICs (SACTA 2: *C*max = 0.72, 31.58% inhibition; SACTA 3: *C*max = 0.61, 42.47% inhibition). The greater resilience observed in these communities is likely associated with their hight taxonomic diversity and functional redundancy, which may provide increased resistance to chemical stressors. These findings suggest that SACTAs may pose a lower ecological risk than conventional ABX treatments under environmentally relevant conditions.

Exposure to SACTAs also reduced microbial metabolic activity, particularly the utilisation of polymers and carbohydrates, and was accompanied by partial growth inhibition. These results highlight the importance of evaluating interactions between phytochemicals and ABXs in complex biological systems, where ecological responses may differ substantially from those observed in laboratory-based single-species assays.

Overall, these findings suggest that SACTAs could reduce antibiotic inputs and associated environmental selection pressure without a proportional increase in toxicity at the riverine microbial-community level, indicating potential environmental advantages. However, the higher sensitivity of *D. magna* and *A. fischeri* to certain formulations, particularly SACTA 3, underscores the need for formulation-specific environmental risk assessments rather than assuming uniform safety across all terpineol–antibiotic combinations.

By combining originality, safety, and a marked reduction in antibiotic use with the potential to mitigate resistance, SACTAs build on existing approved antibiotics rather than discarding the substantial investment behind them. Future work should prioritise the development of stable, safe formulations. Before clinical application, comprehensive safety assessments including in vitro cytotoxicity and genotoxicity assays and short-term in vivo studies will be required to define safe exposure ranges. Subsequent pilot studies in topical antimicrobials, cosmetic preservatives or medicated feeds could then evaluate their efficacy and resistance-selection potential compared with conventional antibiotic monotherapy, supporting their translation into sustainable, antibiotic-sparing alternatives.

## Figures and Tables

**Table 1:** Acute toxicity assays on individual bioindicators. Median effective concentrations (EC_50_, μg/mL) for *Daphnia magna* and *Aliivibrio fischeri* exposed to TER and ABXs. The 95% confidence intervals (CI) are indicated in parenthesis.

Compound	*Daphnia magna*		*Aliivibrio fischeri*
	*EC*_50_ 24 h (95% CI)	*EC*_50_ 48 h (95% CI)	*EC*_50_ 30 min (95% CI)
TER	51.23 (44.66–57.49)	21.493 (16.71–26.70)	17.654 (15.95–19.49)
GTM	840.75 (742.70–927.24)	687.04 (626.07–743.50)	>1000
CHL	285.63 (235.12–333.92)	157.04 (127.75–191.51)	197.25 (171.57–225.51)
TC	391.07 (352.71–430.33)	232.93 (189.94–285.95)	n.d.
FF	326.40 (263.50–406.30)	220.14 (176.79–274.02)	>1000

n.d.: not determined.

**Table 2:** SACTAs toxicity, as *EC*_50_, on *Daphnia magna* and *Allivibrio fischeri* expressed as a percentage and in μg/mL of the mixture. In all cases, the 95% confidence intervals are indicated in parenthesis. For each Synergistic Antimicrobial Combinations of terpineol and antibiotics (SACTAs), the composition in μg/mL is indicated. The Effect column indicates the sort of interaction between SACTAs components. *EC*_50_ values (expressed as a percentage of the total concentration of SACTAs) were compared using paired Holm-Šídák tests on estimates derived from the model. Values that do not share a superscript letter differ significantly (Holm-Šídák-adjusted *p* < 0.001).

		*EC*_50_ *D. magna* 24 h		*EC*_50_ *D. magna* 48 h			*EC*_50_ *A. fischeri* 30 min		
SACTA	Composition	%	µg/mL	%	µg/mL	Effect	%	µg/mL	Effect
1	TER 125 + GTM 3.9	38.74 (31.85–47.49)	48.43 (39.81–59.37) + 1.51 (1.24–1.85)	20.56 **^A^** (12.81–28.45)	25.70 (16.01–35.56) + 0.80 (0.50 + 1.10)	AD	38.78 **^A^** (36.79–40.82)	48.48 (45.99–51.03) + 1.51 (1.43–1.59)	n.d.
2	TER 62.5 + GTM 3.9	82.05 (67.61–100.48)	51.28 (42.26–62.80) + 3.20 (2.64–3.92)	50.59 **^B^** (42.34–60.24)	31.62 (26.46–37.65) + 1.97 (1.65–2.35)	AT	67.12 **^B^** (61.41–72.85)	41.95 (38.38–45.53) + 2.62 (2.39–2.84)	n.d.
3	TER 125 + CHL 15.6	29.88 (24.08–37.00)	37.35 (30.10–46.25) + 4.66 (3.76–5.77)	6.27 **^C^** (2.00–11.44)	7.84 (2.50–14.30) + 0.98 (0.31–1.78)	SY	10.20 **^C^** (8.77–11.78)	12.75 (10.96–14.73) + 1.59 (1.37–1.84)	SY
4	TER 125 + TC 15.6	46.52 (37.73–58.82)	58.15 (47.16–73.53) + 7.26 (5.89–9.18)	20.86 **^A^** (16.18–25.89)	26.08 (20.23–32.36) + 3.25 (2.52–4.04)	AD	n.d.	n.d.	n.d.
5	TER 62.5 + FF 7.8	94.95 (78.23–117.47)	59.34 (48.89–73.42) + 7.4 (6.10–9.16)	51.96 **^B^** (43.05–62.47)	32.48 (26.91–39.04) + 4.05 (3.36–4.87)	AT	59.90 **^B^** (54.12–65.71)	37.44 (33.8–41.07) + 4.67 (4.22–5.13)	n.d.

AD: Additive; AT: Antagonistic; SY: Synergistic; n.d.: not determined.

**Table 3:** Estimated values of the carrying capacity (*C_max_*) and the intrinsic growth rate (*r*) obtained by fitting *AWCD* values to Equation (6) (see Section 3.5.1 for details). The table also includes the percentage of inhibition relative to the control for TER and antibiotics (ABXs), applied either individually or combined within SACTAs.

	μg/mL	*C_max_*	*r*	Inhibition (%)
Control	n.a.	1.06	0.04	n.a.
TER	500	0.24	0.04	77.33
	250	1.01	0.02	5.09
GTM	15.6	0.44	0.03	58.38
	3.9	1.02	0.04	3.93
TC	62.5	0.58	0.01	44.79
	15.6	0.20	0.03	81.50
CHL	125	0.32	0.03	69.55
	15.6	0.89	0.04	16.38
FF	31.5	0.36	0.05	65.97
	7.8	0.75	0.05	28.83
SACTA 1	(*)	0.43	0.03	59.64
SACTA 2	(*)	0.72	0.05	31.58
SACTA 3	(*)	0.61	0.04	42.47
SACTA 4	(*)	0.44	0.01	58.36
SACTA 5	(*)	0.26	0.06	75.15

n.a.: Not applicable; (*): For further details, see Table 4.

**Table 4:** Composition list of the different synergistic antimicrobial combinations of TER and ABX (SACTA) assayed in this study, along with their antimicrobial targets and their individual minimum inhibitory concentration (MIC) values, as previously described by Valenzuela et al. [6].

ID	Components	[TER] (µg/mL)	[ABX] (µg/mL)	Bacterial Target	TER MIC (µg/mL)	ABX MIC (µg/mL)
SACTA1	TER + GTM	125	3.9	*Staphylococcus aureus* ATCC 9144 *Streptococcus agalactiae* ATCC 12386	500	15.6
SACTA2	TER + GTM	62.5	3.9	*Acinetobacter baumanii* ATCC 19606	250	15.6
SACTA3	TER + CHL	125	15.6	*Serratia marcescens* ATCC 13880	500	125
SACTA4	TER + TC	125	15.6	*Serratia marcescens* ATCC 13880	500	62.5
SACTA5	TER + FF	62.5	7.8	*Staphylococcus aureus* ATCC 9144	500	31.25

## Data Availability

The original contributions presented in the study are included in the article and Supplementary Materials. Further inquiries can be directed to the corresponding authors.

## Supplementary Materials

The following supporting information can be downloaded at https://www.mdpi.com/article/10.3390/antibiotics15090929/s1. Figure S1: Metabarcoding complete report; Figures S2: Biolog Ecoplate^TM^ metabolic profiling of SACTA 1; Figure S3: Biolog Ecoplate^TM^ metabolic profiling of SACTA 2; Figure S4: Biolog Ecoplate^TM^ metabolic profiling of SACTA 3; Figure S5: Biolog Ecoplate^TM^ metabolic profiling of SACTA 4; Figure S6: Biolog Ecoplate^TM^ metabolic profiling of SACTA 5, Table S1: Logit parameters for terpineol and antibiotics on individual bioindicators, Table S2: Logit parameters for synergistic antimicrobial combinations of terpineol and antibiotics (SACTAs) on individual bioindicators, Table S3: Physicochemical properties of terpineol and antibiotics used in this study. Table S4, physicochemical analysis of river sample. References [138,139,140,141,142,143] are cited in the Supplementary Materials.

## Abbreviations

The following abbreviations are used in this manuscript:

AWCD	Average well colour development
CHL	Chloramphenicol
*CLPP*	Community-level physiological profile
FF	Florfenicol
GTM	Gentamicin
MIC	Minimum inhibitory concentration
SACTA	Synergistic antimicrobial combinations of terpineol-antibiotic
TC	Tetracycline
