# Supplementary material for: Exploring α-Terpineol–Antibiotic Synergistic Combinations as an Environmentally Safer Antimicrobial Strategy for Freshwater Systems
Source: Antibiotics (Basel). 2026 Sep 19;15(9):929. doi: 10.3390/antibiotics15090929 (PMC13603864; doi:10.3390/antibiotics15090929)
Supplement: Supplementary file 1 [file antibiotics-15-00929-s001.zip › 1 Figure S1_Metabarcoding complete report.pdf]

16S Metagenomics Report

Sample: RPA-04

16S Metagenomics Labs, Version: 1.0.0

Report Date: 06/21/2024 08:40:27

Figure S1: Metabarcoding complete report

Sample Configuration

Sample ID:	RPA-04
Sample Name:	RPA-04
Run Folder:	/data/scratch/workspace/RunFolder
Taxonomy File:	gg_13_5_species_32bp.dat

Sample Information

Total Reads	Reads Passing Quality Filtering	% Reads Passing Quality Filtering
0	303,953	Infinity

Classification Statistics

Taxonomic Level	Reads Classified to Taxonomic Level	% Total Reads Classified to Taxonomic Level
Kingdom	303,789	99.95 %
Phylum	302,886	99.65 %
Class	301,945	99.34 %
Order	300,421	98.84 %
Family	295,797	97.32 %
Genus	284,555	93.62 %
Species	73,147	24.07 %

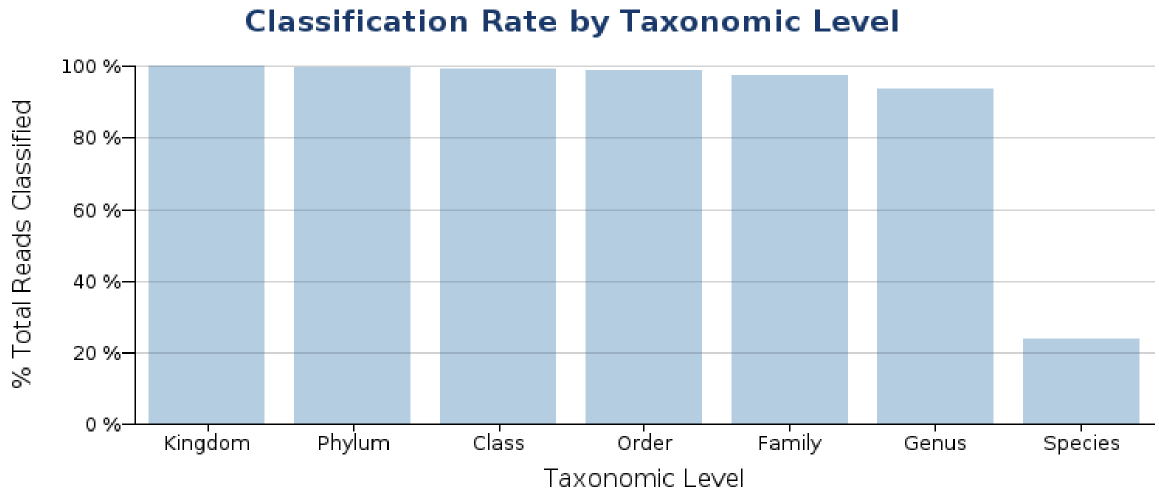

Sunburst Classification Chart

This sunburst chart shows the relative abundance of the classification results within each taxonomic level.
Click on any category to zoom in. Click the center button to zoom out.
Click "Reset Plot" to return to a Kingdom-level view.

Reset Plot

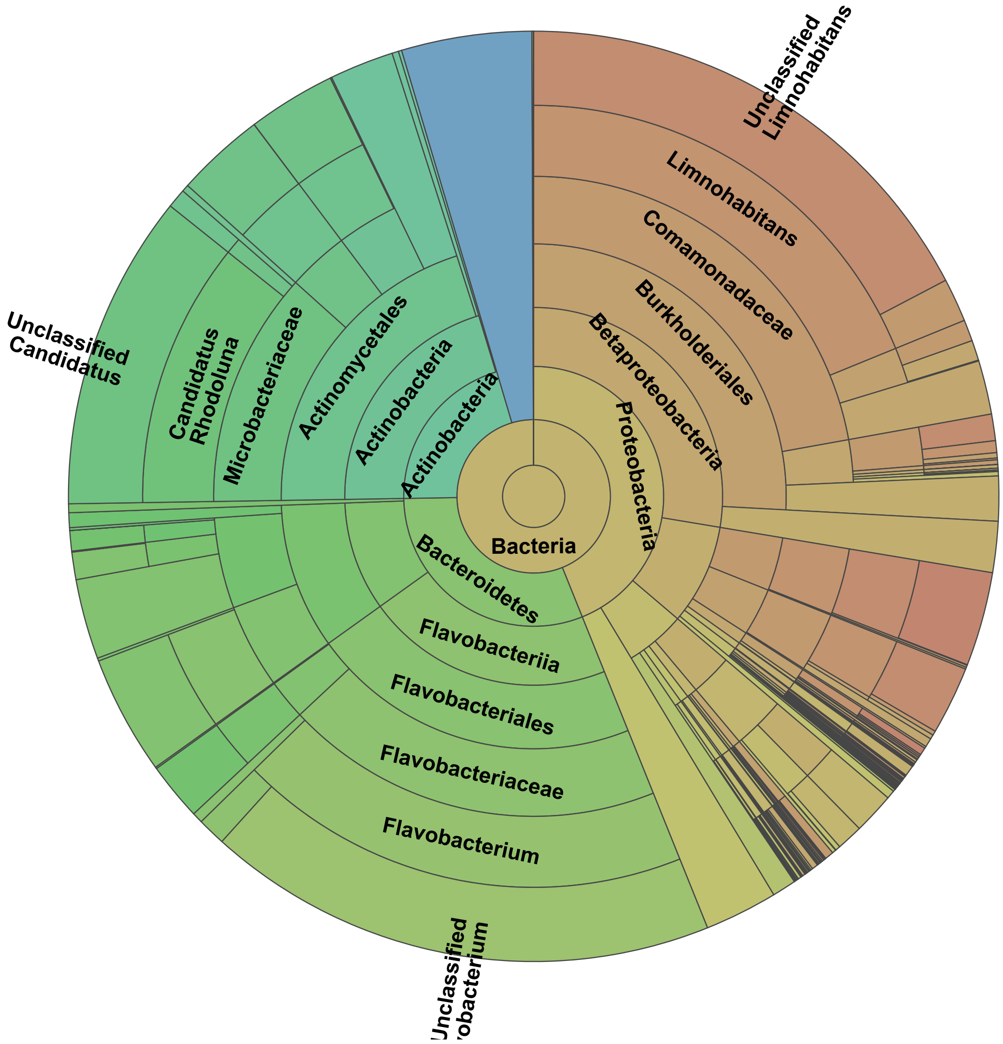

Top 20 Classification Results by Taxonomic Level

This bar chart shows the relative abundance of the top 20 classification results within each taxonomic level.
Mouse over any category to see its description and abundance.

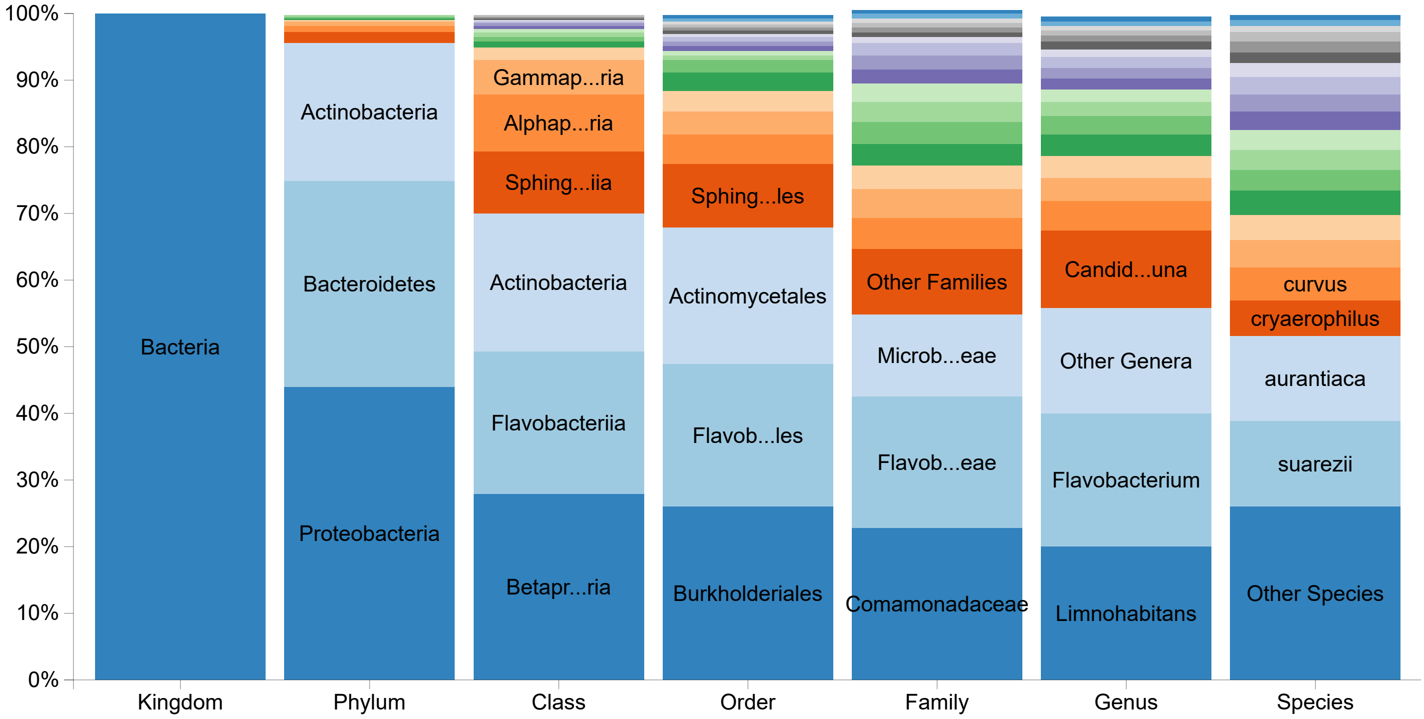

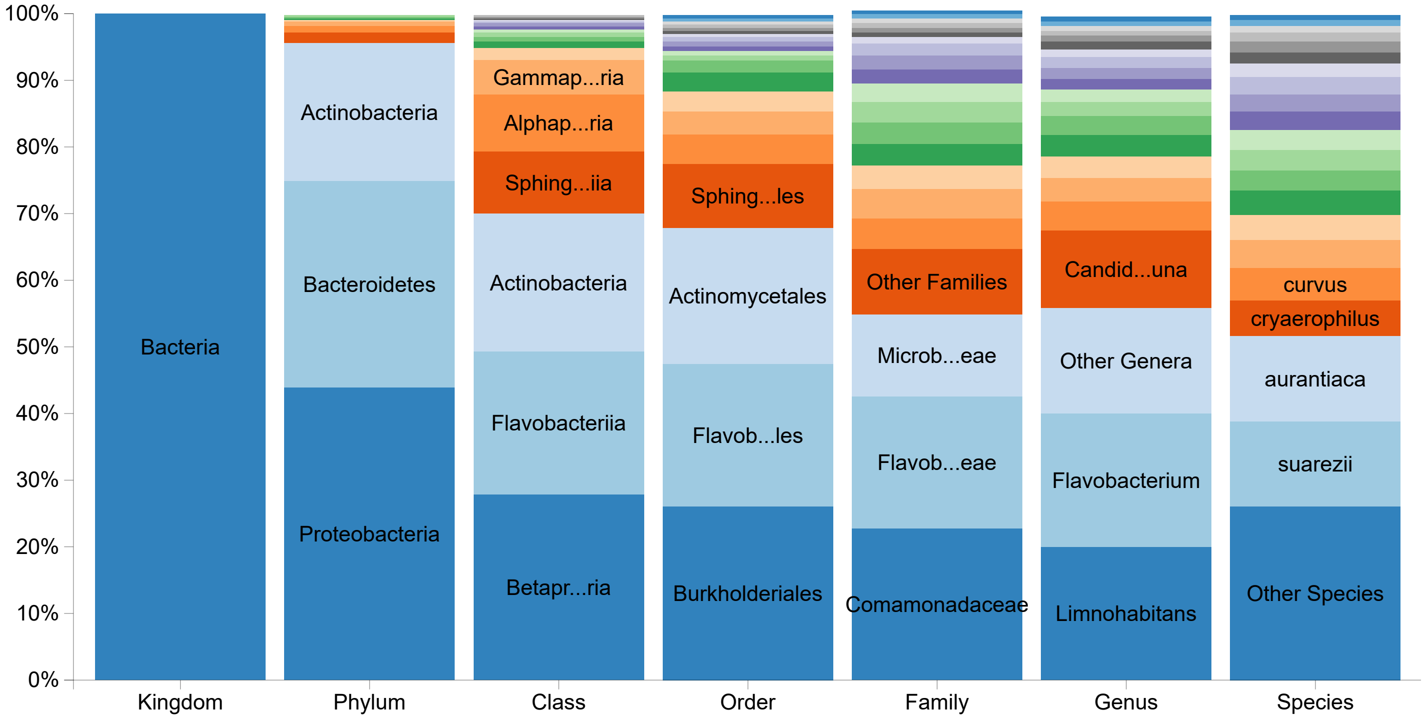

Classification Results by Taxonomic Level

Tables show the highest 8 taxonomic classifications at each level.
Pie charts show all classifications above 3.5% abundance.

Top Kingdom Classification Results

Classification	Number of Reads	% Total Reads
Bacteria	303,764	99.94 %
Unclassified at Kingdom level	164	0.05 %

Archaea	21	0.01 %
Viruses	4	0.00 %

Note: The "Other" category in this pie chart is the sum of all classifications with less than 3.50 % abundance.

Top Kingdom Classification Results

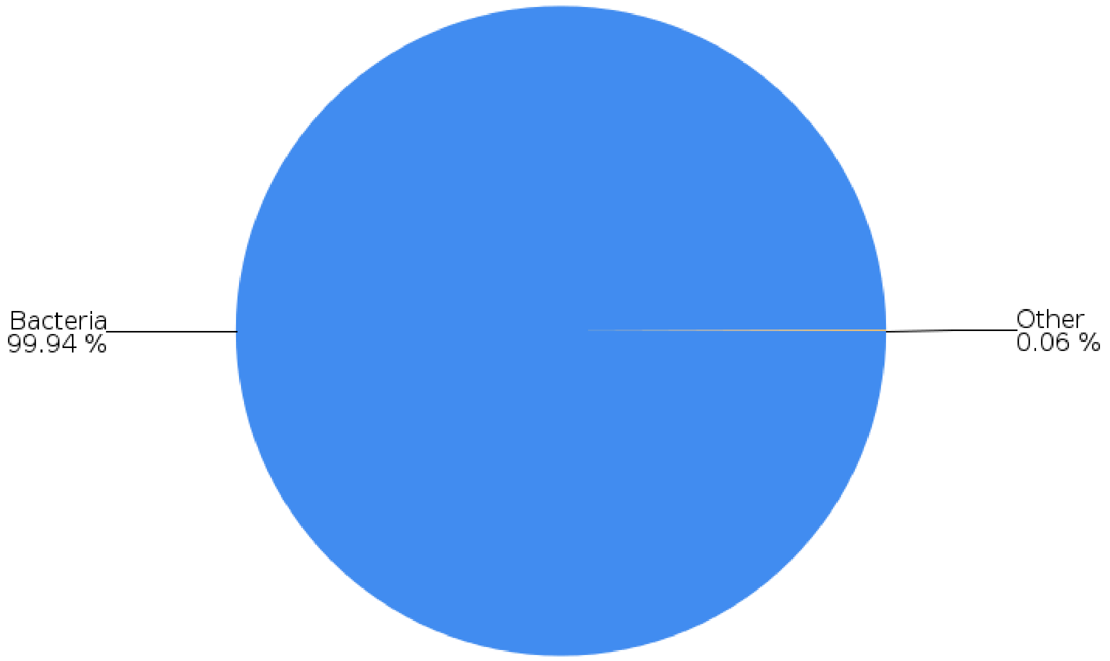

Top Phylum Classification Results

Classification	Number of Reads	% Total Reads
Proteobacteria	133,450	43.90 %
Bacteroidetes	93,739	30.84 %
Actinobacteria	62,836	20.67 %
Firmicutes	4,707	1.55 %
Cyanobacteria	2,742	0.90 %
Verrucomicrobia	2,010	0.66 %
Unclassified at Phylum level	1,067	0.35 %
Planctomycetes	928	0.31 %

Total Phylum-level Taxonomic Categories Identified: 29. This table shows the top 8 of 29 classifications.

Note: The "Other" category in this pie chart is the sum of all classifications with less than 3.50 % abundance.

Top Phylum Classification Results

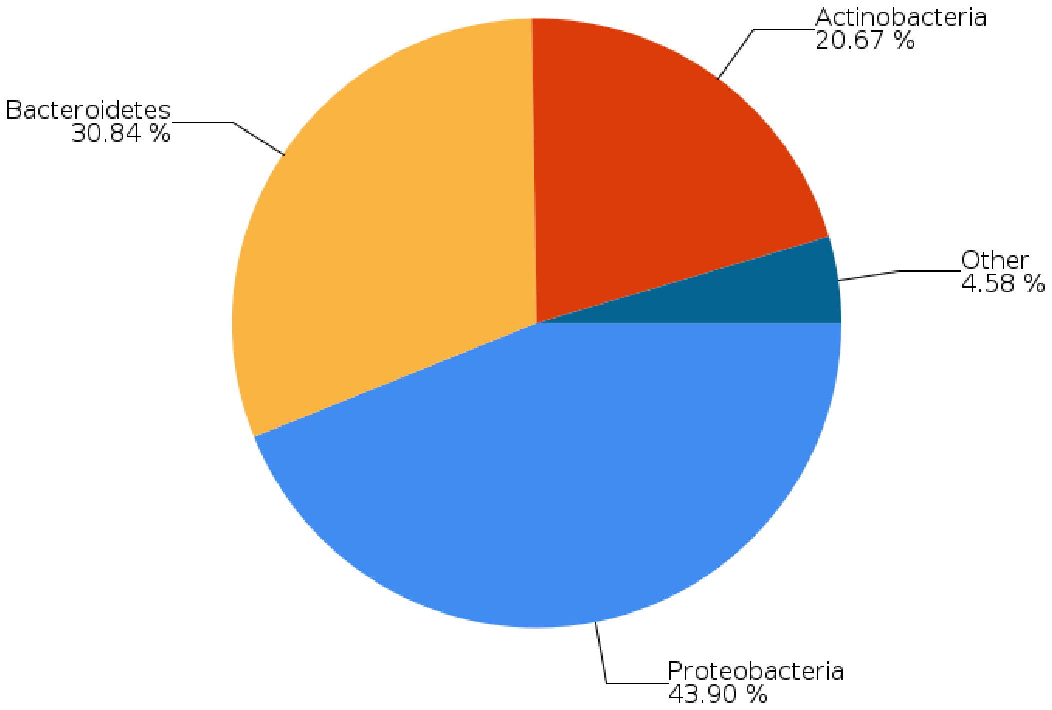

Top Class Classification Results

Classification	Number of Reads	% Total Reads
Betaproteobacteria	83,977	27.63 %
Flavobacteriia	64,438	21.20 %
Actinobacteria	62,493	20.56 %
Sphingobacteriia	28,369	9.33 %
Alphaproteobacteria	26,124	8.59 %
Gammaproteobacteria	15,644	5.15 %
Epsilonproteobacteria	5,617	1.85 %
Bacilli	2,707	0.89 %

Total Class-level Taxonomic Categories Identified: 56. This table shows the top 8 of 56 classifications.
Note: The "Other" category in this pie chart is the sum of all classifications with less than 3.50 % abundance.

Top Class Classification Results

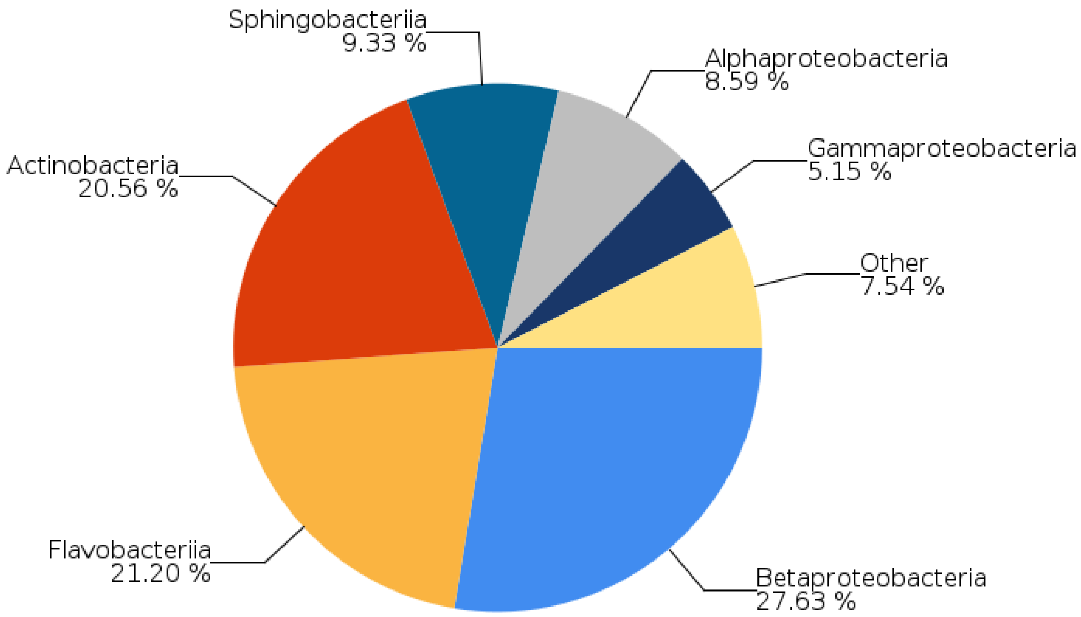

Top Order Classification Results

Classification	Number of Reads	% Total Reads
Burkholderiales	78,586	25.85 %
Flavobacteriales	64,438	21.20 %
Actinomycetales	61,807	20.33 %
Sphingobacteriales	28,369	9.33 %
Rhodobacterales	10,419	3.43 %
Sphingomonadales	9,274	3.05 %
Enterobacteriales	8,115	2.67 %
Campylobacterales	5,617	1.85 %

Total Order-level Taxonomic Categories Identified: 106. This table shows the top 8 of 106 classifications.
Note: The "Other" category in this pie chart is the sum of all classifications with less than 3.50 % abundance.

Top Order Classification Results

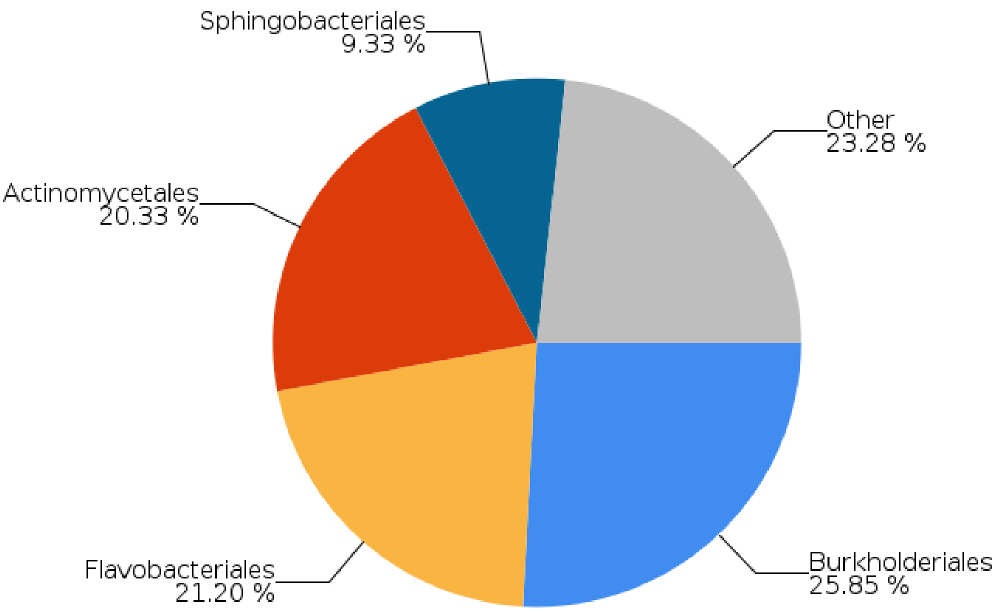

Top Family Classification Results

Classification	Number of Reads	% Total Reads
Comamonadaceae	67,370	22.16 %
Flavobacteriaceae	58,176	19.14 %
Microbacteriaceae	36,183	11.90 %
Chitinophagaceae	13,924	4.58 %
Flexibacteraceae	12,890	4.24 %
Rhodobacteraceae	10,225	3.36 %
Cellulomonadaceae	9,444	3.11 %
Sanguibacteraceae	9,408	3.10 %

Total Family-level Taxonomic Categories Identified: 221. This table shows the top 8 of 221 classifications.
Note: The "Other" category in this pie chart is the sum of all classifications with less than 3.50 % abundance.

Top Family Classification Results

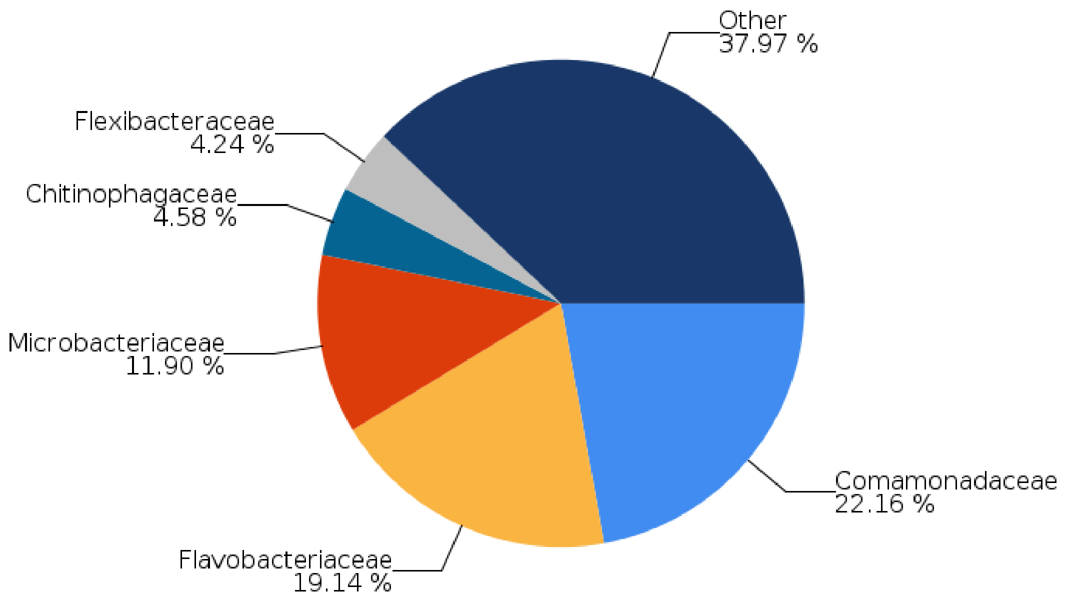

Top Genus Classification Results

Classification	Number of Reads	% Total Reads
Limnohabitans	57,223	18.83 %
Flavobacterium	57,048	18.77 %
Candidatus Rhodoluna	33,395	10.99 %
Unclassified at Genus level	19,398	6.38 %
Arcicella	12,575	4.14 %
Rhodobacter	9,994	3.29 %
Sanguibacter	9,408	3.10 %
Demequina	9,303	3.06 %

Total Genus-level Taxonomic Categories Identified: 582. This table shows the top 8 of 582 classifications.

Note: The "Other" category in this pie chart is the sum of all classifications with less than 3.50 % abundance.

Top Genus Classification Results

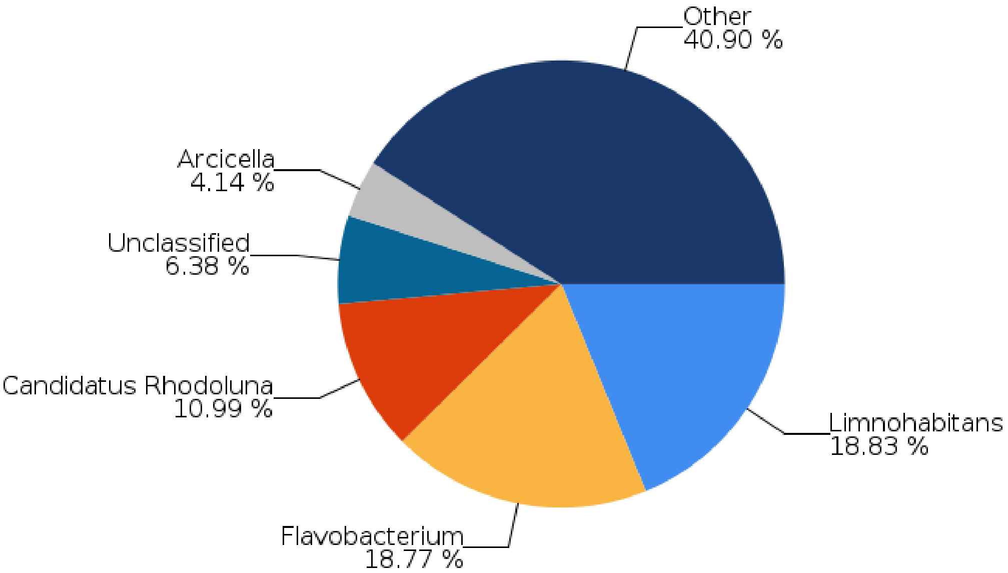

Top Species Classification Results

Classification	Number of Reads	% Total Reads
Unclassified at Species level	230,806	75.93 %
Sanguibacter suarezii	9,395	3.09 %
Demequina aurantiaca	9,284	3.05 %
Arcobacter cryaerophilus	3,976	1.31 %
Limnohabitans curvus	3,539	1.16 %
Chitinophaga soli	2,902	0.95 %
Polynucleobacter necessarius	2,804	0.92 %
Yersinia intermedia	2,783	0.92 %

Total Species-level Taxonomic Categories Identified: 862. This table shows the top 8 of 862 classifications.
Note: The "Other" category in this pie chart is the sum of all classifications with less than 3.50 % abundance.

Top Species Classification Results

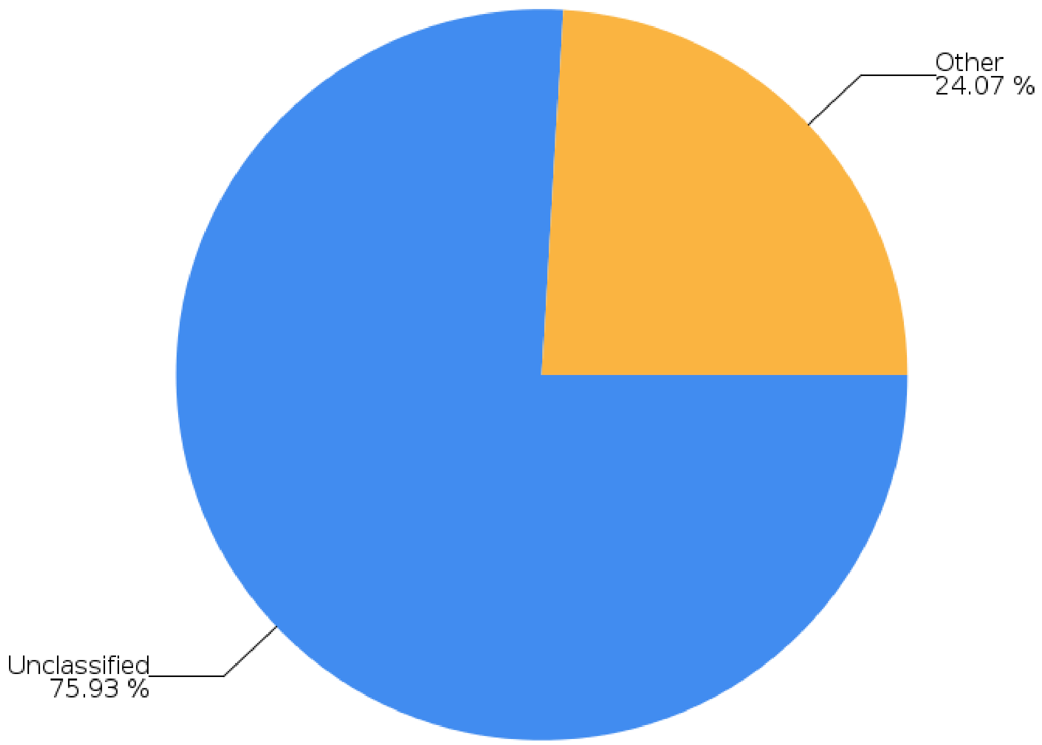

© 2024 Illumina, Inc. All rights reserved.
