# Supplementary material for: Exploring α-Terpineol–Antibiotic Synergistic Combinations as an Environmentally Safer Antimicrobial Strategy for Freshwater Systems
Source: Antibiotics (Basel). 2026 Sep 19;15(9):929. doi: 10.3390/antibiotics15090929 (PMC13603864; doi:10.3390/antibiotics15090929)
Supplement: Supplementary file 1 [file antibiotics-15-00929-s001.zip › 2 Figures S2-S6_SACTAs CLPP Complete.pdf]

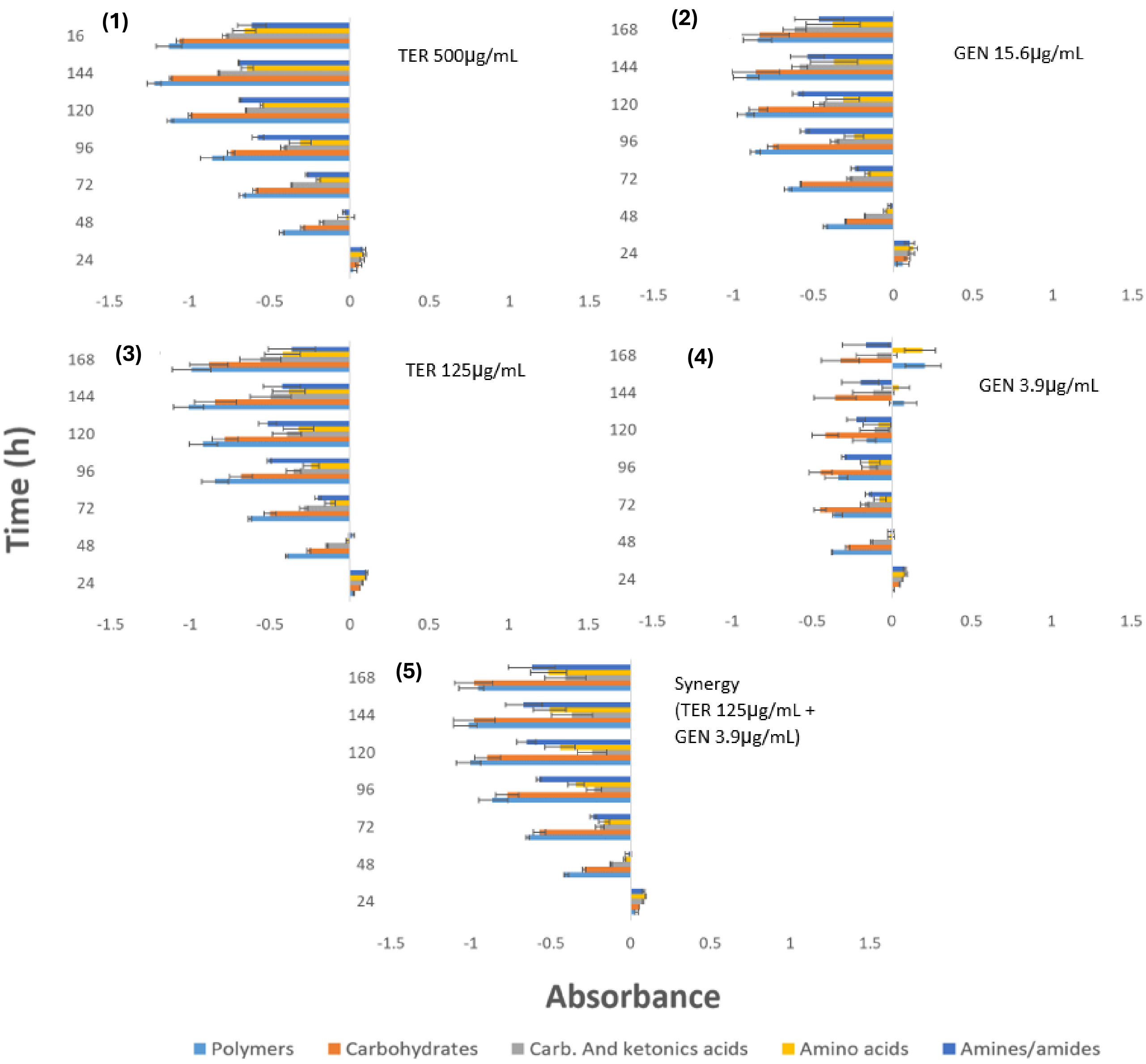


**Figure S2.** Biolog Ecoplate™ metabolic profiling of SACTA 1.

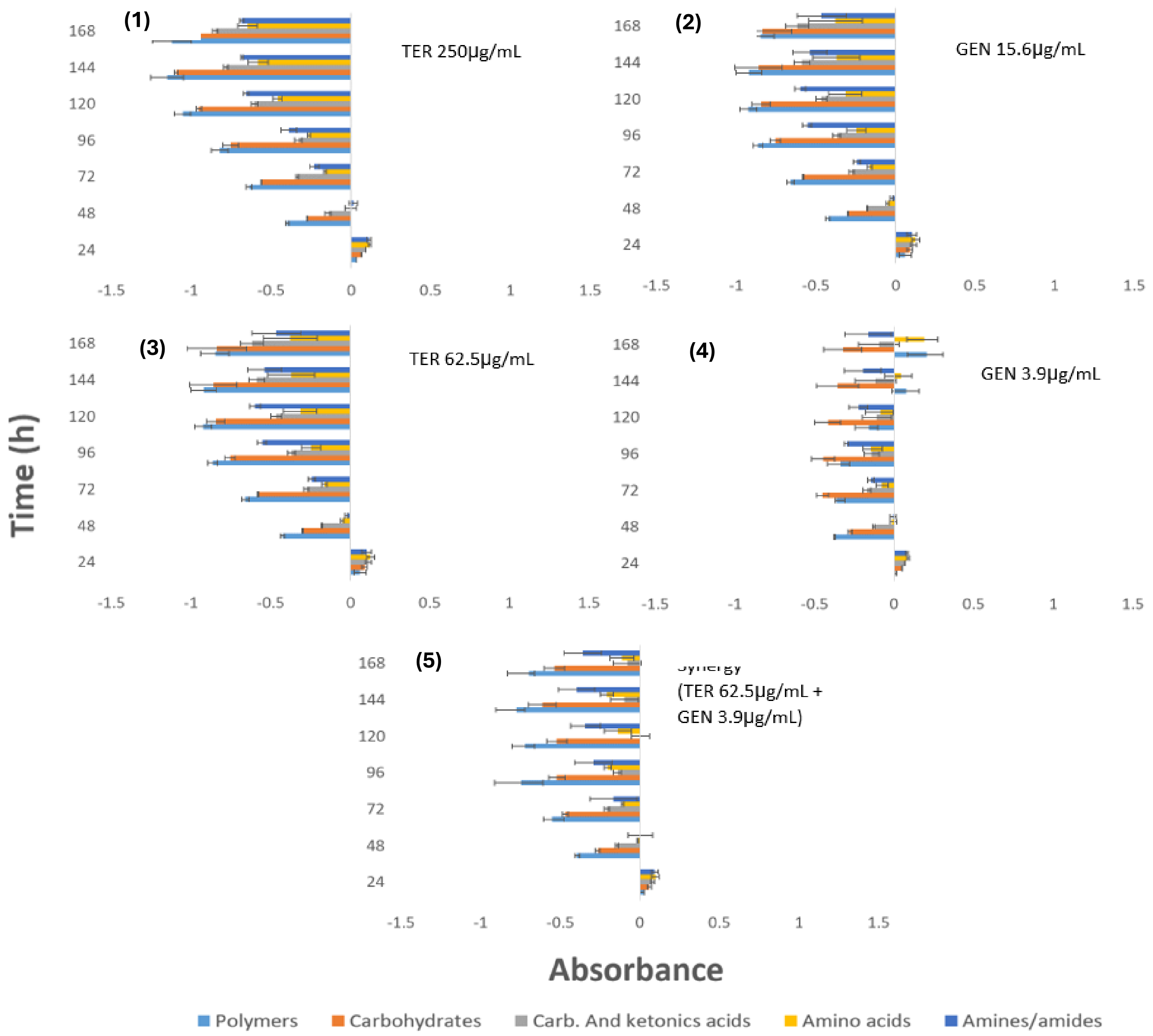


**Figure S3.** Biolog Ecoplate™ metabolic profiling of SACTA 2.

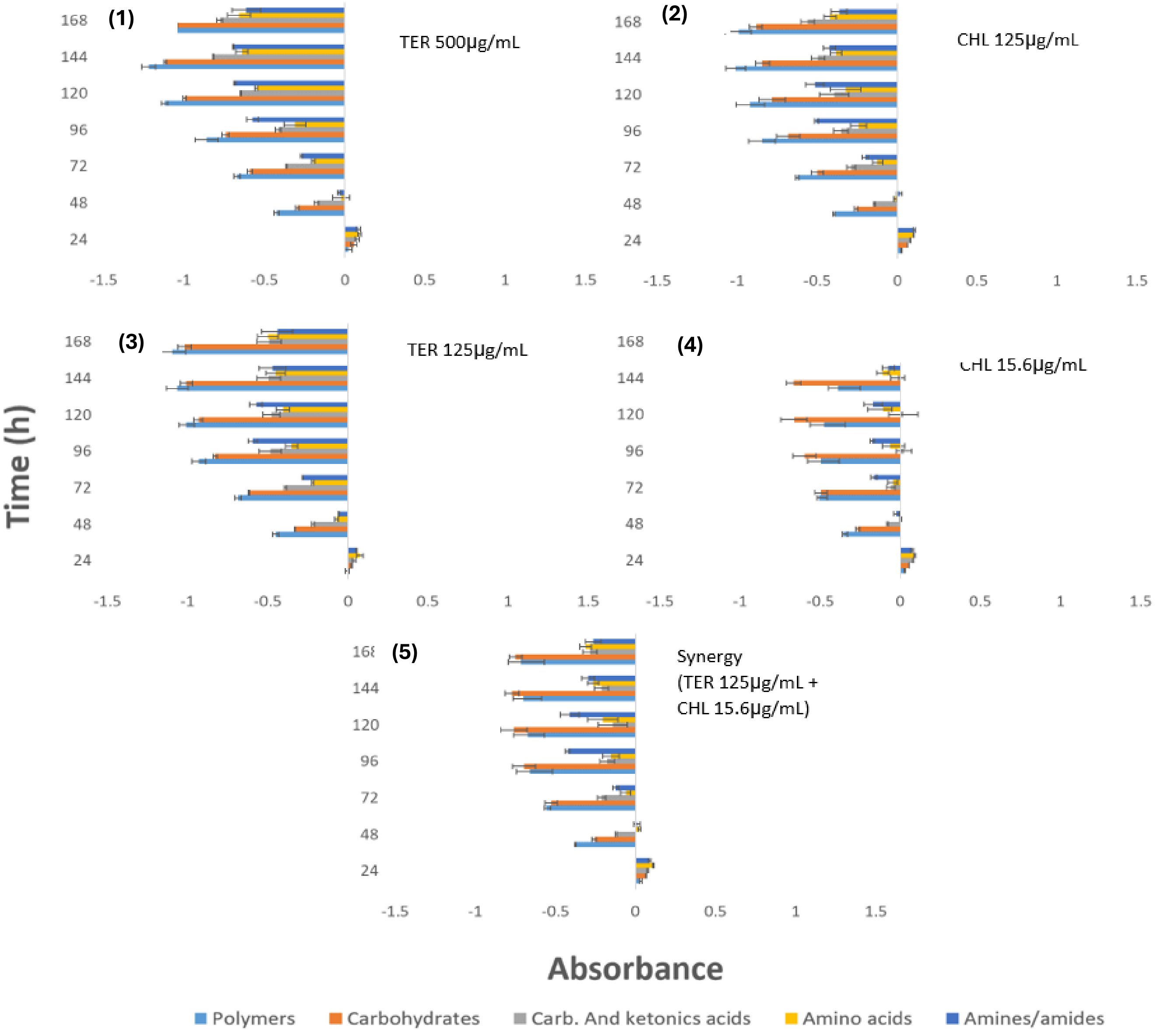


**Figure S4.** Biolog Ecoplate™ metabolic profiling of SACTA 3.

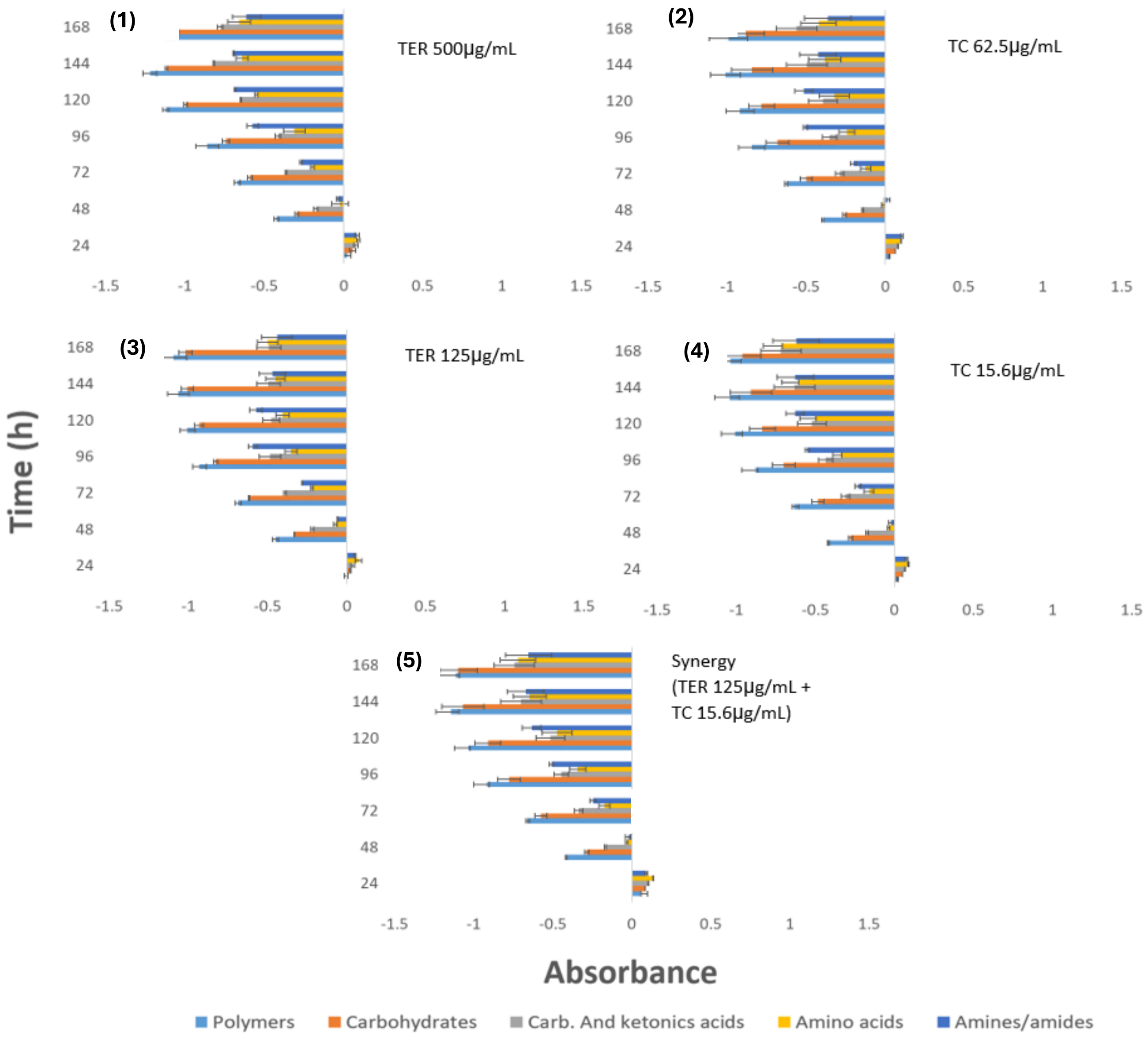


**Figure S5.** Biolog Ecoplate™ metabolic profiling of SACTA 4.

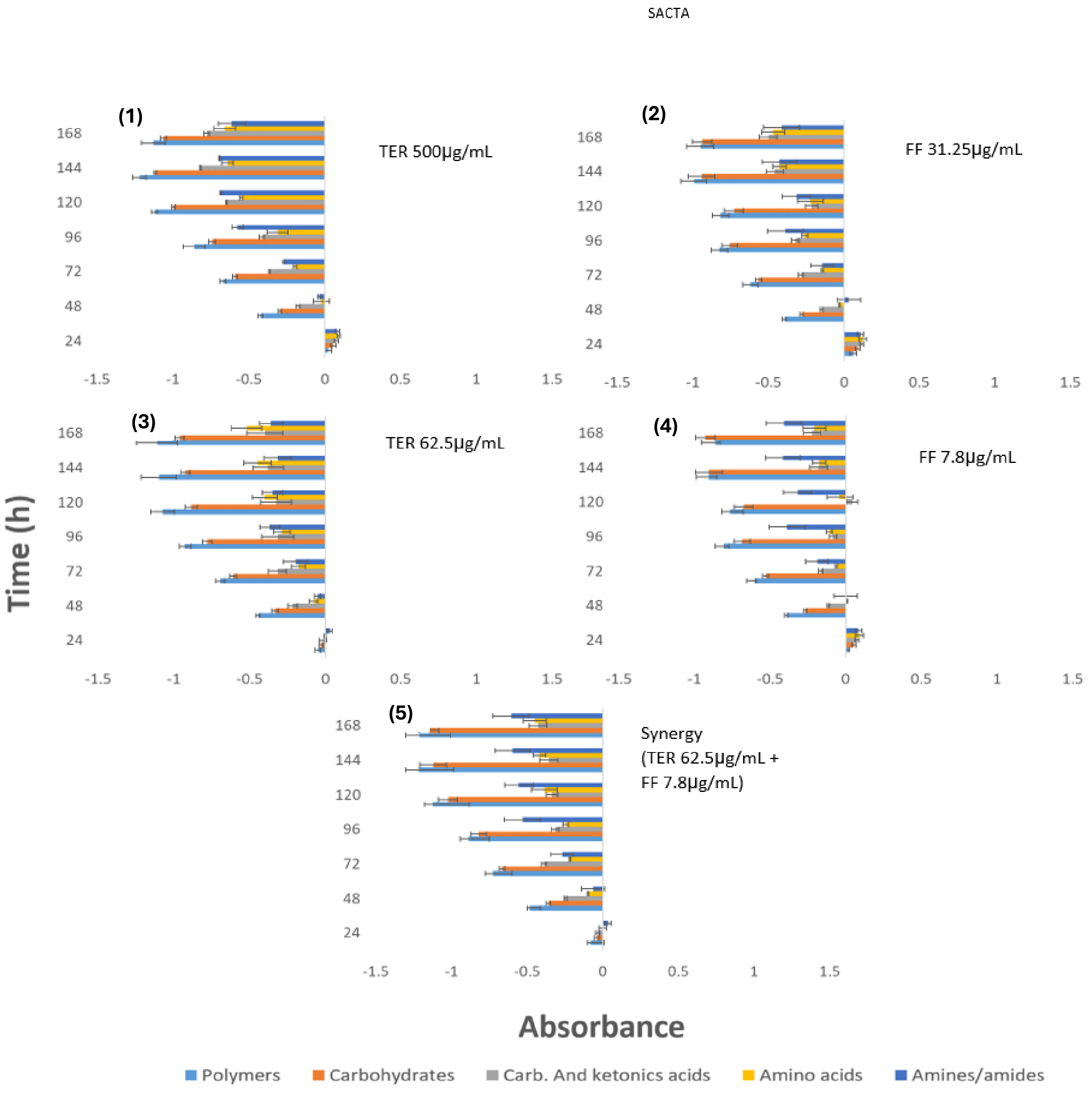


**Figure S6.** Biolog Ecoplate™ metabolic profiling of SACTA 5.
