# Supplementary material for: Exploring α-Terpineol–Antibiotic Synergistic Combinations as an Environmentally Safer Antimicrobial Strategy for Freshwater Systems
Source: Antibiotics (Basel). 2026 Sep 19;15(9):929. doi: 10.3390/antibiotics15090929 (PMC13603864; doi:10.3390/antibiotics15090929)
Supplement: Supplementary file 1 [file antibiotics-15-00929-s001.zip › 3 Tables S1-S3 def.pdf]

Table S1: Logit parameters for terpineol and antibiotics on individual bioindicators.

ID	*D. magna* (24 h)				*D. magna* (48 h)				*A. fischeri* (30 min)			
	β_0	β_1	*LR* (X^2)	*p*	β_0	β_1	*LR* (X^2)	*p*	β_0	β_1	*LR* (X^2)	*p*
Terpineol	−18.731	10.057	80.540	<0.0001	−8.058	6.048	88.374	<0.0001	−5.510	4.418	1598-901	<0.0001
Gentamicin	−39.942	13.907	70.341	<0.0001	−10.211	6.494	86.754	<0.0001	n.d.	n.d.	n.d.	n.d.
Tetracycline	−37.380	14.560	73.600	<0.0001	−15.910	6.721	87.456	<0.0001	n.d.	n.d.	n.d.	n.d.
Chloramphenicol	−32.006	11.936	82.818	<0.0001	−15.354	6.992	87.419	<0.0001	0.891	1.345	129.328	<0.0001
Florfenicol	−15.231	6.059	79.237	<0.0001	−13.834	5.905	78.785	<0.0001	n.d.	n.d.	n.d.	n.d.

Table S2: Logit parameters for synergistic antimicrobial combinations of terpineol and antibiotics (SACTAs) on individual bioindicators.

ID	*D. magna* (24 h)				*D. magna* (48 h)				*A. fischeri* (30 min)			
	β_0	β_1	*LR* (X^2)	*p*	β_0	β_1	*LR* (X^2)	*p*	β_0	β_1	*LR* (X^2)	*p*
SACTA 1	-11.852	7.462	86.933	<0.0001	−4.495	3.424	63.628	<0.0001	−10.613	6.681	1367.543	<0.0001
SACTA 2	−14.611	7.634	85.883	<0.0001	−15.894	3.303	108.567	<0.0001	−6.827	3.737	1150.532	<0.0001
SACTA 3	−9.582	6.494	88.754	<0.0001	−1.705	2.139	62.989	<0.0001	−2.204	2.185	1211.422	<0.0001
SACTA 4	−10.804	6.479	72,161	<0.0001	−8.038	6.093	89.082	<0.0001	n.d.	n.d.	n.d.	n.d.
SACTA 5	−14.853	7.511	78.438	<0.0001	−14.430	8.411	103.199	<0.0001	−5.89	3.314	1151.778	<0.0001

Table S3: Physicochemical properties of terpineol and antibiotics used in this study.

Family	Compound	CAS No.	Molecular weight (g/mol)	Purity	Water solubility (mg/mL)	pKa1/pKa2	Log Ko/w	References
Terpenes	Terpineol	8000-41-7	154.3	≤ 100%	2.54	19.4/−0.87	2.98	[1,2]
Aminoglycosides	Gentamycin	1403-66-3	447.6	≥ 97%	12.6	12.6/10.1	−1.88	[3]
Tetracyclines	Tetracycline	64-75-5	444.4	99.20%	2.31	7.68/3.3	1.37	[4]
Amphenicols	Chloramphenicol	56-75-7	323.1	97.50%	2.5	8.7/−2.8	1.14	[4,5]
	Florfenicol	73231-34-2	358.2	-	9.94	10.73	−0.04	[6,7]

References

1. Merck Terpineol Available online: https://www.sigmaaldrich.com/ES/es/product/aldrich/86480?srsltid=AfmBOoqxtrjbsNJLZzMCtuEmTjHhNuXfFaGLODmGImGq9xamdeZi9Jmc (accessed on 26 March 2026).
2. Li, J.; Perdue, E.M.; Pavlostathis, S.G.; Araujo, R. Physicochemical Properties of Selected Monoterpenes. *Environ. Int.* **1998**, *24*, 353–358, doi:10.1016/S0160-4120(98)00013-0.
3. Chemical Book Gentamicin Available online: https://www.chemicalbook.com/ChemicalProductProperty_EN_CB7308330.htm (accessed on 26 April 2026).
4. Hansch, Corwin.; Leo, Albert.; Hoekman, D.H.. *Exploring QSAR: Hydrophobic, Electronic, and Steric Constants*; American Chemical Society, 1995; ISBN 0841229937.
5. O'Neil, M.J. *The Merck Index : An Encyclopedia of Chemicals, Drugs, and Biologicals*; Royal Society of Chemistry, 2013; ISBN 9781849736701.
6. Chan, R.; Wandee, S.; Wang, M.; Chiemchaisri, W.; Chiemchaisri, C.; Yoshimura, C. Fate, Transport and Ecological Risk of Antibiotics from Pig Farms along the Bang Pakong River, Thailand. *Agric. Ecosyst. Environ.* **2020**, *304*, 107123, doi:10.1016/J.AGEE.2020.107123.
7. Settimo, L.; Bellman, K.; Knegtel, R.M.A. Comparison of the Accuracy of Experimental and Predicted PKa Values of Basic and Acidic Compounds. *Pharm. Res.* **2013**, *31*, 1082–1095, doi:10.1007/S11095-013-1232-Z.
