# Supplementary material for: Exploring α-Terpineol–Antibiotic Synergistic Combinations as an Environmentally Safer Antimicrobial Strategy for Freshwater Systems
Source: Antibiotics (Basel). 2026 Sep 19;15(9):929. doi: 10.3390/antibiotics15090929 (PMC13603864; doi:10.3390/antibiotics15090929)
Supplement: Supplementary file 1 [file antibiotics-15-00929-s001.zip › 4 Table S4. Physicochemical characteristics river water.pdf]

Table S4： physicochemical analysis of river sample

**Supporting Information 2.** Physicochemical parameters of the river water sample collected from the Gállego River (a tributary of the Ebro River; Montañana, Zaragoza, Spain) in May 2024 for water Biolog assays.

| *Parameter* | *Result* | *Unit* |
|---|---|---|
| *MES* | 4.2 | mg/L |
| *Bicarbonate alkalinity* | 0 | mg/L |
| *Total alkalinity* | 246 | mg/L |
| *TDS* | 1239 | mg/L |
| *Ammonium ($NH4^+$)* | <0.1 | mg/L |
| *Chloride ($Cl^-$)* | 381 | mg/L |
| *Sulfate ($SO_4^{-2}$)* | 194 | mg/L |
| *Nitrate ($NO_3^-$)* | 7.06 | mg/L |
| *Nitrite ($NO_2^-$)* | <0.05 | mg/L |
| *Fluoride ($F^-$)* | 0.092 | mg/L |
| *Phosphate ($PO_4^{-3}$)* | <0.2 | mg/L |
| *COD ($O_2$)* | <25 | mg $O_2$/L |
| *BOD5* | <5 | mg $O_2$/L |
| *Oxidability ($O_2$)* | 2.2 | mg $O_2$/L |
| *Calcium ($Ca^+$)* | 129 | mg/L |
| *Magnesium ($Mg^+$)* | 19.4 | mg/L |
| *Sodium ($Na^+$)* | 260 | mg/L |
| *Potassium ($K^+$)* | 2.83 | mg/L |

Abbreviations: MES, suspended matter; TDS, total dissolved solids; COD, chemical oxygen demand; BOD*5*, 5-day biochemical oxygen demand.
